# Optimizing Carbon Structures in Laser-Induced Graphene Electrodes Using Design of Experiments for Enhanced Electrochemical Sensing Characteristics

**DOI:** 10.1021/acsami.4c13124

**Published:** 2024-11-14

**Authors:** Fabiane Fantinelli Franco, Muhammad Hassan Malik, Libu Manjakkal, Ali Roshanghias, Cindy J. Smith, Caroline Gauchotte-Lindsay

**Affiliations:** † Water and Environment Group, Infrastructure and Environment Division, James Watt School of Engineering, University of Glasgow, Glasgow G12 8LT, U.K.; ‡ Silicon Austria Laboratories GmbH, Europastrasse 12, A-9524 Villach, Austria; § School of Computing and Engineering & the Built Environment, Edinburgh Napier University, Merchiston Campus, Edinburgh EH10 5DT, U.K.

**Keywords:** screen printing, laser-induced graphene, design of experiments, nitrite detection, graphene and graphitic structures, electrochemical studies

## Abstract

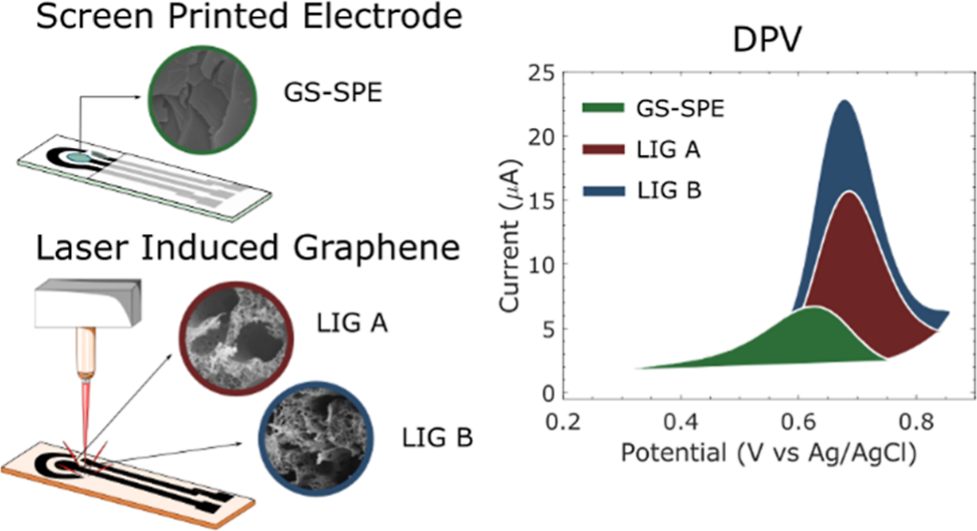

In this study, we explored the morphological and electrochemical properties of carbon-based electrodes derived from laser-induced graphene (LIG) and compared them to commercially available graphene-sheet screen-printed electrodes (GS-SPEs). By optimizing the laser parameters (average laser power, speed, and focus) using a design of experiments response surface (DoE-RS) approach, binder-free LIG electrodes were achieved in a single-step process. Traditional trial-and-error methods can be time-consuming and may not capture the interactions between all variables effectively. To address this, we focused on linear resistance and substrate delamination to streamline the DoE-RS optimization process. Two LIGs, designated LIG A and LIG B, were fabricated using distinct and optimized laser settings, which resulted in a sheet resistance of 25 ± 2 Ω/sq and 21 ± 1 Ω/sq, respectively. These LIGs, characterized by scanning electron microscopy, Raman spectroscopy, and contact angle analysis, exhibited a highly porous morphology with 13% pore coverage and a contact angle <50°, which significantly increased their hydrophilicity when compared to the GS-SPE. For the electrochemical studies, the oxidation of NO_2_^–^ ion by the graphene-based working electrodes was investigated, as it allowed for the direct comparison of the LIGs to the GS-SPE. These included cyclic voltammetry, electrochemical impedance spectroscopy, and differential pulsed voltammetry studies, which revealed that LIG electrodes displayed a remarkable 500% increase in peak current during NO_2_^–^ oxidation compared to the GS-SPE. The LIGs also demonstrated improved stability and sensitivity (420 ± 30 and 570 ± 10 nAμM^–1^ cm^–2^) compared to the GS-SPE (73 ± 4 nAμM^–1^ cm^–2^) in the oxidation of NO_2_^–^ ions; however, LIG B was more susceptible to ionic interference than LIG A. These findings highlight the value of applying statistical approaches such as DoE-RS to systematically improve the LIG fabrication process, enabling the rapid production of optimized LIGs that outperform conventional carbon-based electrodes.

## 1 Introduction

Graphene and graphene-based materials have been incorporated into various areas of technology since their discovery due to their high conductivity, specific surface area, thermal conductivity, and tensile strength.^1^ In order to be successfully employed in industry, various fabrication routes have been proposed to scale the production of graphene, including chemical-vapor deposition, liquid-phase exfoliation, and reduction to graphene oxide; however, they tend to be cumbersome and expensive.^1^ Nowadays, graphene-based pastes and inks have been implemented in printed electronics as an alternative to metal-based conductive tracks.^2,3^ This allows for a more sustainable approach to printed electronics. In this regard, screen-printing is an established fabrication method due to its scalability, reliability, and usage of inexpensive materials.^4^ In screen-printed electrodes (SPEs), carbon is an inexpensive and inert material that is typically used in the active working electrode (WE) area of electrochemical sensors. It is not usually employed on the conductive tracks because of its lower conductivity; however, additives such as carbon nanotubes or graphene flakes have been shown to decrease the SPE resistance.^5−7^ The problem is that binders and solvents are necessary in the paste formulation; therefore, the quantity of graphene or other additives is limited.

**Figure 1:**
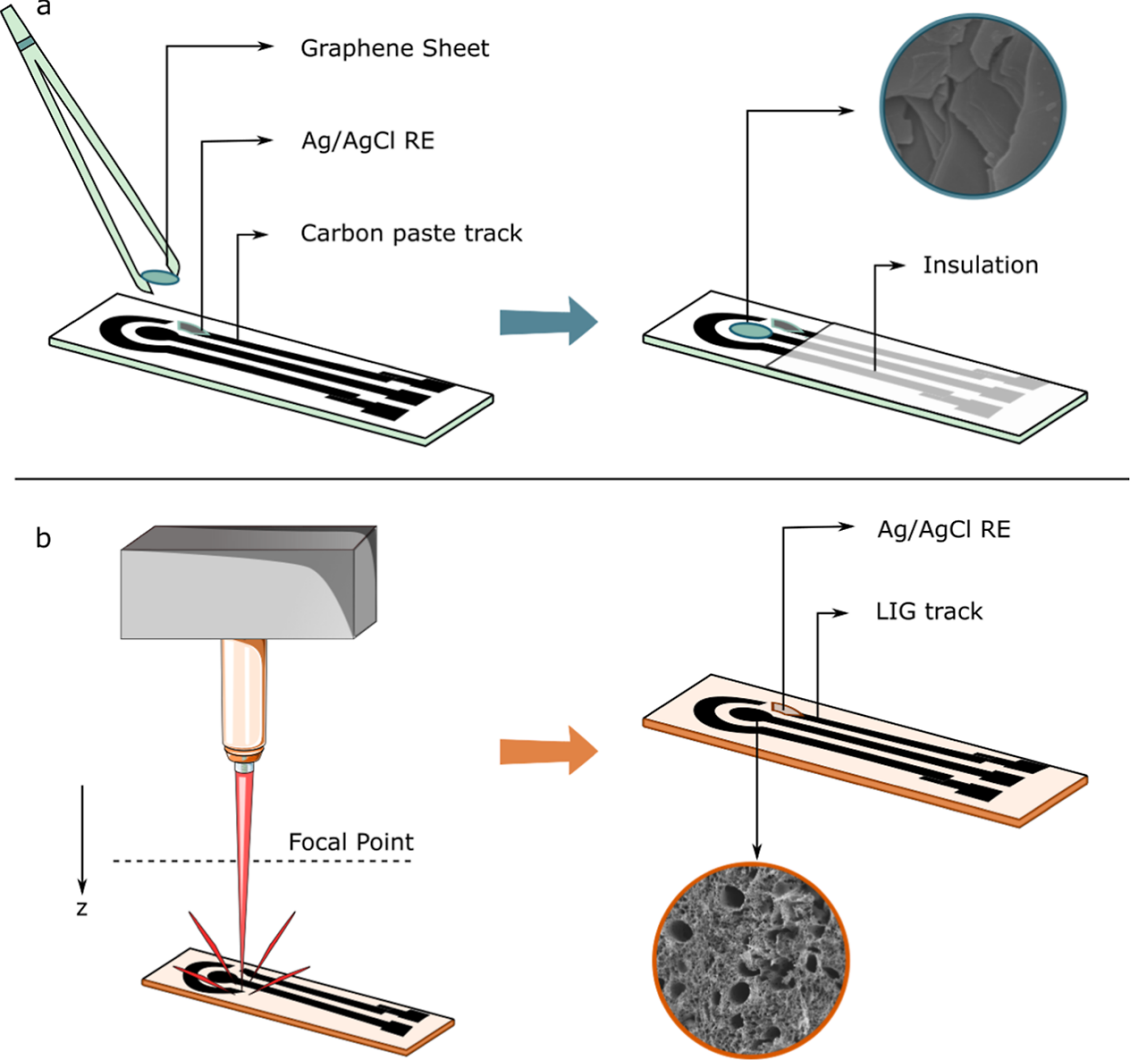
(a) Fabrication steps of the graphene-sheet screen-printed electrodes (GS-SPEs). The graphene sheet was attached to the commercial carbon WE. An insulation layer protects the screen-printed carbon track. The scanning electron microscopy (SEM) inset of the graphene sheet shows smooth graphene layers. (b) Fabrication steps of the LIG electrodes. Laser engraving on the polyimide substrate was done by adjusting from the focal point in the positive *z* direction and changing the average laser power and speed. For the final electrode, Ag/AgCl paste was drop-casted on the printed electrode to form the reference electrode (RE). The SEM inset shows the 3D porous carbon network of the LIG.

A new method to engrave three-dimensional graphene-like structures called laser-induced graphene (LIG) was developed by Lin et al.^8^ The local high temperature combined with a high-pressure environment generated by the CO_2_ laser allowed for the graphitization of the polyimide (PI) substrate. The breakage of C–O, C=O, and C–N bonds of the PI produces high-pressure gas pockets.^9^ This facilitates the formation of nanopores, micropores, and other defects, creating a porous 3D graphene-based network that cannot be achieved through other techniques.^9,10^ For instance, LIGs (Figure 1b) achieve a complex 3D network compared to the relatively smooth surface of graphene sheets (Figure 1a). PI is a thermally stable substrate but sensitive to alkali conditions, which could affect its long-term durability and application range.^11,12^ Although PI is the most used substrate for the formation of LIG, the technique allows for the usage of any source of carbon-based feedstock material, e.g., wood, textile, charcoal, and even food items, which allows LIGs with different properties to be fabricated.^9,13^ For instance, the use of polybenzoxazine as a precursor has shown promise due to its high thermal stability, superior chemical resistance, and high adhesion, which contribute to enhanced durability.^12^ More environmentally friendly substrates can also be used as a precursor to LIG. For example, a cellulose filter paper sprayed with a fire retardant has been shown to produce LIGs with good electrical conductivity and sheet resistance of 32 Ω/sq.^14^ Another substrate used was chitosan cross-linked with borax that produced LIG electrodes with sheet resistances as low as 110 Ω/sq.^15^

Therefore, improving the stability of LIG electrodes can be achieved by optimizing both the precursor materials and the fabrication processes. However, as LIG is a relatively new technology that was first published in 2014, the key parameters influencing the formation of high-quality graphene-like structures, as well as their mechanical, electrochemical stability, and conductivity, can still be tailored and optimized for specific applications.^8^ To help the process of LIG optimization, one-factor-at-a-time (OFAT) approaches are commonly used, where one parameter, such as average laser power or speed, is changed successively until a desired outcome is reached.^16^ Although this is a valid technique, utilizing other statistical approaches can be more advantageous, as they can save time and material resources when compared to OFAT. Design of experiments (DoE) is a resource-efficient optimization process that can minimize the cost of the experiment by creating models to indicate where the minimum/maximum desired output is.^17^ This approach can simplify the optimization of LIGs, as complex systems can be analyzed using less samples than the OFAT alternative as it reduces the number of experiments required.^18,19^ Furthermore, another important aspect of DoE is the ability to highlight parameter interdependency, which is unlikely to be determined when using OFAT approaches. This is particularly advantageous for LIG fabrication, as it is a new technique that can benefit from optimization strategies. The morphology and performance of LIG electrodes are highly responsive to minor variations in laser processing parameters, such as laser power, speed, and focus, and number of laser passes.^18,19^ Previous DoE studies on LIG fabrication optimization focused on comparing variable responses such as sheet resistance, cyclic voltammetry (CV), peak current, and Raman spectra.^18−20^ These response variables can provide valuable insights into the LIG quality. An additional response variable that is not commonly assessed is the adhesion of LIG to the substrate. For fast design of experiments response surface (DoE-RS) results, assessing both the LIG resistance and substrate adhesion can provide a swift and effective evaluation of the LIG performance. Once an area of optimal laser parameters has been found, further rounds of DoE-RS can be performed, and more specialized response variables can be included, such as Raman spectra and electrode activity.

When LIGs are compared to SPEs, many factors can be considered. For example, LIGs offer a template-free method to produce highly conductive graphene-based electrodes, while SPEs require the use of a mask, which can limit the final pattern resolution.^21^ However, screen-printing is a mature, versatile technology, with the possibility of combining various conductive pastes to produce the final product, while LIGs are confined to carbon structures.^4,22^ Both techniques have similarities, including a low production cost linked to a high yield. This information is summarized in Table 1. LIG and SPEs have been employed in numerous fields, including supercapacitors, solar cells, fuel cells, and electrochemical sensors.^4,9,23−25^ LIGs are especially interesting in the field of electrochemical sensors due to their high surface area and high conductivity, as this allows for efficient modification of the WE with various materials and faster electron transfer. For example, LIG electrodes could offer advantages in the detection of NO_2_^–^, as this ion benefits from fast detection techniques due to its short environmental life and it can be detected using unmodified carbon materials.^26^

**Table 1:** Comparison of Screen-Printing and Laser Engraving Techniques for Electrode Fabrication

	screen printing	LIG
pattern	requires a mask (e.g., stainless steel)	mask-free
resolution	40 μm	12 μm
sheet resistance	<5 mΩ/sq for Ag paste< 35 Ω/sq for carbon paste	<30 Ω/sq in polyimide
production	high yield, fast speed	high yield, fast speed
substrate	any compatible with the paste used	carbon-based precursors
materials	metal, carbon, and insulating pastes	carbon structures from the substrate
commercial cost	<£1/sensor (e.g., DropSens)	<£2/sensor (e.g., Gii-Sens)
further modifications	WE and RE can be screen-printed using different pastes	requires other techniques to modify WE and RE
availability	commercially available	mostly research-based

Increasing levels of nitrite (NO_2_^–^) and nitrate (NO_3_^–^) in ground and surface water have been detected as a result of excessive usage of nitrogen fertilizers, runoff waste from livestock farms, and use as a food preservative.^27,28^ In the environment, high concentration of nitrogenous compounds can lead to eutrophication of water bodies.^28^ In humans, oxygen transportation can be hindered by the NO_2_^–^ ion by the irreversible conversion of hemoglobin to methemoglobin, which is particularly problematic for pregnant women and infants.^29,30^ Due to its toxic nature, the recommended levels of NO_2_^–^ in drinking water are below 3 mg/L.^29,31^ As NO_2_^–^ is a reactive ion, the detection techniques need to provide a fast, accurate result. However, most of the standard instrumentation involves the use of expensive, time-consuming methods, such as spectrophotometric, chemiluminescence, spectrofluorometric, and ion chromatography detection.^28,30,32^ Recently, electrochemical methods have also been employed for the NO_2_^–^ detection, focusing on potentiometric and voltammetric approaches; however, they are not as sensitive as standard techniques.^28^ The fast analysis time counterbalances the higher detection limit, with voltammetric and amperometric techniques being the fastest electrochemical methods. Considering that the NO_2_^–^ ion is highly reactive, a fast technique is very advantageous. Regarding the choice of the sensitive material, carbon-based electrodes are widely used for the electrochemical detection of NO_2_^–^ due to their ability to oxidize NO_2_^–^ to NO_3_^–^, but they are usually modified with other metal/metal oxides for higher sensitivity.^33^ Common materials include gold, copper, and iron nanoparticles, and the study is mostly done using standard laboratory electrodes, such as the glassy-carbon WE.^33,34^ Carbon-based printed/engraved sensors, such as carbon-based SPEs and LIGs, can be a good alternative to standard electrodes as they can be readily employed in the field.^35,36^

In this work, we investigated how the material morphology of carbon-based electrodes affects their electrochemical properties by comparing LIGs optimized using a DoE approach to a standard commercial SPE. The aim is to understand if LIGs are a suitable replacement for SPEs, as they provide a low-cost, template-free alternative to carbon SPEs. For this, we used a commercial SPE with graphene/carbon-based tracks and a multilayer graphene sheet (MGS) on top of the WE (GS-SPE) (Figure 1). Meanwhile, the LIGs were engraved on PI and were optimized using a DoE response surface (DoE-RS) approach. By changing different parameters (average laser power, speed, and focus) that affect the carbonization of the PI substrate, two optimized LIGs were chosen to continue the experiments, LIG A and LIG B. Both the GS-SPE and the LIGs were employed without further modification on all studies, simplifying their use. The material characterization was done by SEM, Raman spectroscopy, and contact angle analysis. For the electrochemical studies, the oxidation of NO_2_^–^ ion by the graphene-based working electrodes was investigated, as it allowed for the direct comparison of the LIGs to the GS-SPE. The electrochemical investigation was done by CV, electrochemical impedance spectroscopy (EIS), and differential pulsed voltammetry (DPV).

## 2 Experimental Section

### 2.1 Electrode Fabrication

#### 2.1.12.1.1 Screen-Printed Electrode

In this work, a commercial SPE was acquired from JE Solutions with 45 mm length by 1.5 mm width conductive tracks and a circular WE of 3 mm diameter. The total size of the SPE was 45 mm in length by 6 mm in width. The conductive tracks were screen-printed using a graphene conductive paste on top of a poly(ethylene terephthalate) (PET) substrate. The RE was screen-printed using silver/silver chloride (Ag/AgCl) paste. The conductive tracks were covered with white thermoplastic polyurethane insulation ink, leaving only the WE, RE, and counter electrode (CE) exposed. In the laboratory, a commercial pristine MGS (Graphene Supermarket, 25 μm thickness) was cut into a 3 mm diameter circle and bonded on top of the WE with graphene paste (JE Solutions). The GS-SPE was then cured at 80 °C for 30 min and used in further experiments. The GS-SPE is seen in Figure 1.

#### 2.1.22.1.2 Laser-Induced Graphene Electrodes

A Universal Laser Systems PLS6150D CO_2_ laser engraver with 10.6 μm wavelength and 0–75 W average laser power (also referred to as power instead of average laser power throughout the text) was used for the LIG engraving. This laser system manages the average laser power by controlling the exposure time and pulse density, allowing for precise control over the engraving. The max speeds on the *x*-axis and *y*-axis were 1778 and 508 mm/s, respectively. The electrode pattern was done in Inkscape by setting the outline to 0.001 mm and filling the pattern with a color for the laser system software (Universal Laser Systems software) to follow. The sensor design had a 20 mm length by 1.5 mm width conductive tracks and a circular WE of 3 mm diameter, the same design employed in the SPEs. The total size of the three-electrode system was 24 mm length by 9 mm width. For the DoE, only the WE was fabricated and once the system was optimized, the three-electrode LIGs were used for further material and electrochemical characterization. A polyimide substrate (PI, 500HN Kapton film from Utech Products, 127 μm thickness) was selected for the LIG fabrication. The high proportion of aromatic rings present in the PI substrate is ideal for the graphitization process and the formation of a porous 3D structure.^37^ The Kapton film with a 127 μm thickness presented a good compromise between substrate thickness and flexibility. The PI substrate was adhered to a plastic substrate using deionized water for a residue-free bond. First, a water droplet was added on top of the plastic layer, and the PI substrate was then positioned on top. The surface tension of the water prevents the PI substrate from moving during the engraving but also allows it to be easily removed from the plastic substrate once the laser process is done. For the graphitization of the substrate, the PI was patterned under ambient conditions using the Universal Laser Systems software. The focus was adjusted by first adjusting the laser to the focal point and then using software to increase or decrease the distance to the substrate. Ag/AgCl paster (JE Solutions) was drop-cast on top of the LIG RE, and the final electrode can be seen in Figure 1. The Ag/AgCl paste was used to minimize external factors that could contribute to the difference in WE performance, as the aim of the paper is to compare the carbon materials used in the WE. For other purposes, the Ag/AgCl RE is not necessarily needed, and it would be interesting to study the effect of unmodified LIG as a RE.

### 2.22.2 Design of Experiments

The central-composite DoE-response surface (DoE-RS) method was employed to find the best performing LIGs by analyzing which combination of parameters used during fabrication give the best results. An important part is to correctly select the parameters that most affect the results and the initial range of values to be used. These were selected based on the reported literature values where graphitization can occur.^16,18^ To optimize the LIGs for resistance and adhesion to the PI substrate, DoE-RS was performed iteratively based on both the linear resistance response (Figure S1) and the LIG adhesion to the substrate (delamination). While linear resistance was chosen to simplify the experiments and expedite data collection, measuring sheet resistance offers a more comparable method to literature values. Therefore, sheet resistance of the final LIGs was also measured for comparison. DoE-RS is an advanced DoE technique that allows for the optimization of a response that is influenced by several parameters.^17^ In this way, a sequential procedure is used to determine the optimum operating conditions. For the adhesion response, the number 0 or 1 was allocated to each run, with 0 being no material delamination after the bending process and 1 being full or partial delamination after the substrate. Figure S2 exemplifies the delamination process, where some parameter combinations showed a brittle LIG even before bending. Minitab was used to prepare and analyze all DoE-RS experiments using the central composite design. The linear resistance was taken with a multimeter from the middle of the WE to the middle of the connection pad, while the adhesion was visually inspected by bending the electrode at 180° three times. At first, a 3-parameter, 20-point DoE-RS was performed to select the regions of interest for the next set of DoE-RS (Figure S1). The parameter range was set as follows:

- Speed of 10–30%, corresponding to 178–534 mm/s on the *x*-axis and 51–152 mm/s on the *y*-axis.
- Average laser power of 5–20% corresponding to 3.75–15 W.
- Focus between 0.5 and −1.5 mm.
- Pulse density of 1000 pulses per inch (PPI).

A negative focus value indicates that the substrate is moving closer to the laser. The WE structure was printed twice for each run, and the model was based on the mean linear resistance of the electrodes. Many of the parameters did not graphitize the substrate; therefore, two new two-factor DoE-RS were performed around the regions where graphitization occurred:

i Focus = −1 mm, 10% < Speed <20%, and 10% < Power <20%, PPI = 1000.
ii Focus = −2 mm, 20% < Speed <30%, and 20% < Power <30%, PPI = 1000.

Based on the models, three points of interest were selected to validate each model. The best performing LIG was selected for each DoE-RS, with LIG A consisting of a focus of −1.0 mm, a speed of 20% (356 mm/s on the *x*-axis and 102 mm/s on the *y*-axis), and a power of 10% (7.5 W) and LIG B consisting of a focus of −2.0 mm, a speed of 25% (445 mm/s on the *x*-axis and 127 mm/s on the *y*-axis), and a power of 17.9% (13.5 W).

To summarize the DoE-RS process employed in this paper, a first iteration round was performed using parameters based on fabricated LIGs from literature, which included a selected range of values for laser focus, power, and speed.^16,18^ From literature, a laser density of 500 to 1000 PPI did not significantly alter LIG sheet resistance; therefore, a laser density of 1000 PPI was selected.^18^ The LIG linear resistance and delamination of the LIG from the substrate were the responses of interest for the DoE-RS method as they are important variables to consider when fabricating sensors. Then, a second iteration based on the results of the first iteration was performed to further optimize the LIGs. This round, two constant laser focus values were selected from the regions where graphitization occurred, as changing the focus height is a manual process that can introduce errors to the method. For each focus value, a range of laser speed and power values were chosen based on the best results of the previous DoE-RS iteration. From this second iteration, the best candidate was chosen for each region, resulting in LIG A from region (i) and LIG B from region (ii).

### 2.3 Material Characterization

The resistance of the LIG electrodes for the DoE was measured using a digital multimeter. The resistivity of the electrodes was measured using the Ossila 4-probe system, and by inputting the electrode thickness, length, and width, the sheet resistance was also calculated. The electrodes were characterized by scanning electron microscopy (SEM, FEI QUANTA 200F environmental SEM at 5 kV and aperture size of 30 μm). The ImageJ software was utilized for measuring the height of the cross-section layers and to quantify the LIG pores. For the quantification of the pores, first, the threshold was adjusted and so the pores were highlighted. To decrease the background noise, both the despeckle and remove outlier (<8px for LIG A and <5px for LIG B) features were used. The highlighted area was then analyzed for count and area. The pores were approximated to perfect circles to get the radius. The Raman spectra were obtained using a Renishaw inVia Raman microscope with a 514 nm green Ar laser, an average laser power of 10%, an exposure time of 10 s, a grating of 2400 lines/mm, and an accumulation of 1. The middle of the WE was selected for all Raman spectra, and an extended feature was used to record from 700 to 3200 cm^–1^. After baseline subtraction, the first order (D, G, D′) Lorentzian peaks and second order (2D) peaks were fitted to the spectra using Matlab. The raw Raman plots together with the baseline subtracted ones can be seen in Figure S3. For the contact angle, the Ossila contact angle goniometer was used. 20 μL of deionized water was dropped on top of the electrodes using a syringe and the process was filmed and analyzed by the Ossila contact angle software.

### 2.4 Electrochemical Setup

The CV, DPV, and EIS analysis of the printed electrodes were carried out using the Gamry potentiostat (Interface 1010E). The sensors were attached to an adaptor that is connected to the potentiostat, and the solutions were dropped on top of the WE, RE, and CE (Figure 1). The sensors were washed with Milli-Q water between each experiment. All experiments were done under ambient conditions. A 50 mM NaCl in Milli-Q water solution was used for all electrochemical experiments unless otherwise stated. A concentrated sodium nitrite (NaNO_2_) solution in Milli-Q water was used to change the concentration of NO_2_^–^. The pH was adjusted to 6, 7, or 8 with diluted sodium hydroxide (NaOH) and hydrochloric acid. For the ionic compound interference experiment, 50 μM different ionic compounds in a solution containing 50 μM NaNO_2_ pH 7 were tested one at a time. The ionic compounds were calcium carbonate (CaCO_3_), iron(II) sulfate (FeSO_4_), magnesium sulfate (MgSO_4_), or sodium nitrate (NaNO_3_). Highland spring sparkling water was used for the real water experiment. The GS-SPE and LIG were conditioned by performing five CV scans at 100 mV/s in 50 mM NaCl pH 7. The CVs were scanned from 10, 50 mV/s, to plus 50 mV/s until 250 mV/s with 50 μM NO_2_^–^. The DPVs were run with a step size of 5 mV, a sample period of 0.5 s, a pulse time of 0.2 s, and a pulse size of 50 mV, and the NO_2_^–^ concentration was changed to from 10 to 500 μM. The EIS analysis was obtained from 1 MHz to 0.1 Hz, and the NO_2_^–^ concentration was also changed to from 10 to 500 μM.

## 3 Results and Discussion

### 3.1 Optimization of the Laser-Induced Graphene Electrodes

The LIGs were fabricated using a laser engraver, in which the user can change the power, speed, and focal distance of the laser to graphitize the PI substrate (Figure 1). The combination of these parameters can result in many different possible outputs, making it challenging to obtain a final electrode that is conductive and stable for electrochemical analysis. By employing DoE techniques instead of the usual OFAT experimental approach, the interaction between different factors can be analyzed while greatly reducing the number of experiments needed to produce and identify the best performing LIG.^17^ In the first report of LIG, the authors noted that there was a linear relation between the threshold of the average laser power to the scan speed that led to the graphitization of the substrate.^8^ It has also been observed through other DoE approaches that the power, scan speed, and focus point influence the graphitization of the PI substrate, while a PPI from 500 to 1000 and gas flow did not significantly influence the LIG sheet resistance.^18^ Therefore, the starting set of parameters for the DoE consisted of a three-factor approach, where the focus, speed, and focal point were changed in each run, with 20 runs in total, with 6 of those corresponding to center points. The responses observed initially were linear resistance and visual inspection of the graphitization process. Figure S1 shows a representation of the DoE-RS results, where the regions in which successful graphitization of the material occurred can be observed, e.g., where dark, textured patterns on the PI substrate could be clearly seen. Some of the power, speed, and focus combinations showed no graphitization of the substrate, while others cut through the substrate. Based on the linear resistance of the samples where graphitization did occur, a first DoE-RS model was found. This was then used to narrow the second iteration of the DoE-RS to two most significant factors (speed and power) to achieve a more controlled LIG optimization by analyzing the responses of linear resistance and substrate adhesion.

**Figure 2:**
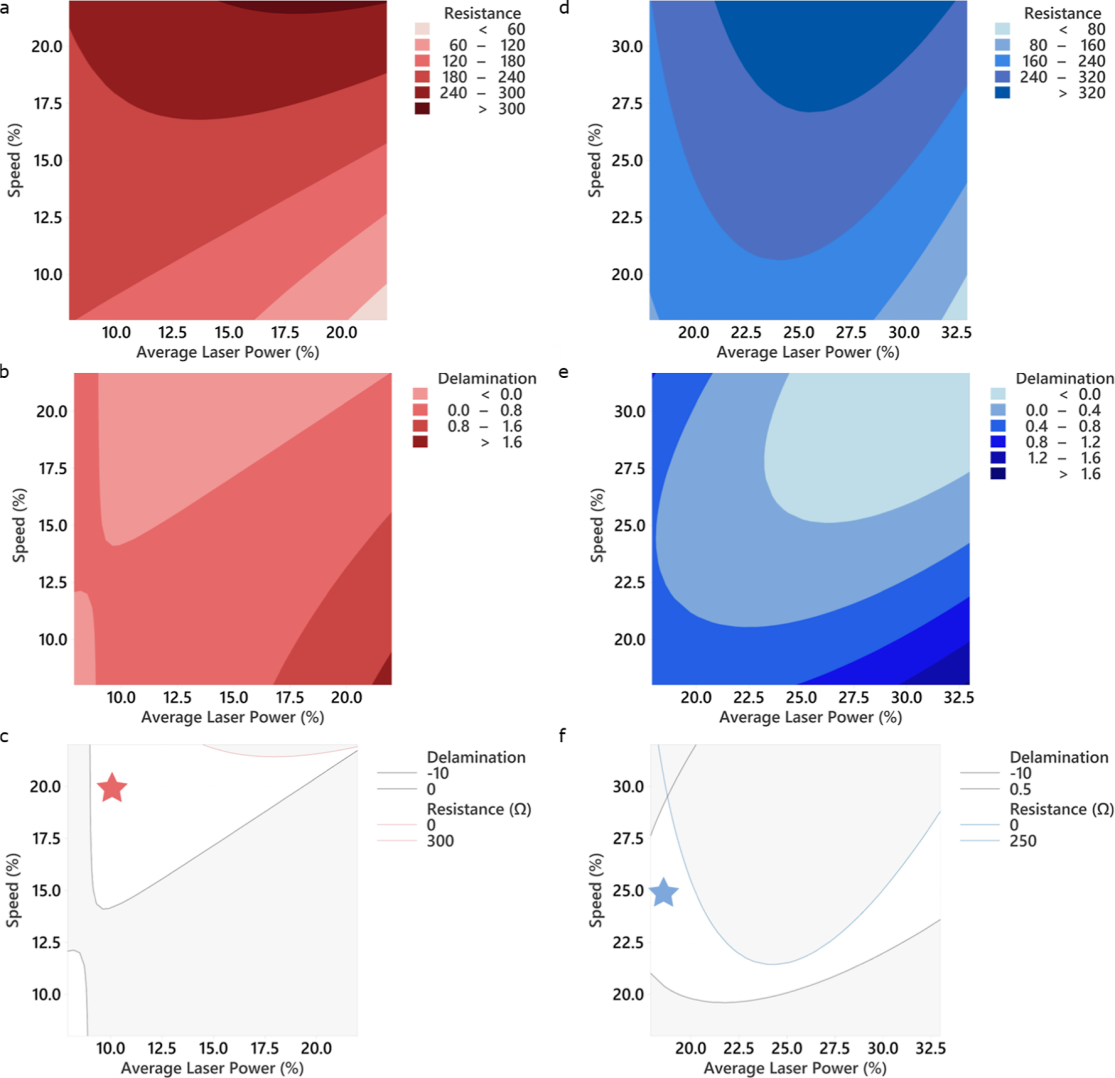
DoE-RS contour plots of engraving speed vs average laser power for the model I (top) and model II (bottom) corresponding to (a,d) linear resistance in Ω of the LIG; (b,e) coded delamination of the electrode, where 0 is no delamination and 1 is full delamination; and (c,f) superposition of the regions with delamination <0 and resistance <300 Ω for model I and resistance <250 Ω for model II. The red star represents the region for LIG A and the blue star the region for LIG B.

For the second iteration of the DoE-RS, two regions were chosen, with model I corresponding to region (i) (Focus = −1 mm, 10% < Speed <20%, and 10% < Power <20%, PPI = 1000) and model II corresponding to region (ii) (Focus = −2 mm, 20% < Speed <30%, and 20% < Power <30%, PPI = 1000). Model I (Table S1) was built from statistically significant terms, showing a good correlation of the given parameters to the resistance model, with a low *p*-value (less than 0.05) for the lack-of-fit parameter (*p*-value of 0.044). While speed showed a linear response to resistance, power demonstrated a quadratic response. Model I presented an overall good fit with a low standard error (Table S2). The diagnostic tests for the validation of the ANOVA test (Figure S4a,c) showed that the data are normally distributed, with random fluctuations of the residuals in the run order. In Figure 2a,c, the DoE-RS for the measured two responses are observed, resistance (Figure 2a) or delamination (Figure 2b) and an overlay of both of these responses for the region of interest (Figure 2c). Although the delamination model was based on 0 being no delamination and 1 visible delamination, the DoE-RS model increased the range of these values to fit the model. The regions with negative values should be understood as no delamination and the regions with a value greater than 1 as full delamination. It is important to note that some regions of low resistance showed some cracks on the surface after bending, making the overlay of these two responses of great importance.

Therefore, to obtain a reliable LIG for further characterization and to validate the model, three regions were chosen for engraving. Table S3 includes the selected power and speed percentages chosen alongside their predicted and measured resistance values. The regions corresponding to a higher speed showed a better correlation to the predicted value, but overall, there was a good fit to the model. The measured resistances were within the 95% confidence and prediction intervals (CI and PI), with the exception of the measured value of 148 Ω using 15% power and speed of 12.5%. The measured value was lower than the predicted value (187 Ω and 158–270 Ω 95% PI), which also corresponded to the lowest speed used. Although this region presented the lowest linear resistance, it also cracked while bending, so it was not suitable for further experiments. Although the resistance model was not as accurate for these power and speed values, this is a region where delamination is more probable to occur, as can be observed in Figure 2c. Therefore, by combining both output models, a better optimization process can be achieved. After this process, the region highlighted with a red star (Figure 2c) was named as LIG A. Various electrodes using the three-electrode configuration were then printed for further material and electrochemical characterization.

The same procedure was repeated using a more defocused region with a focus of −2 mm. The constructed model II (Table S4) showed a *p*-value of 0.075 for the lack-of-fit parameter. This demonstrated that model II is not as robust as model I and needs further adjustments, either by adjusting the model or performing another iteration of DoE. However, it was sufficient for the aim of this study, as it was still possible to obtain a suitable region through the DoE, which would otherwise be difficult to find as the electrodes cracked more often than those from model I. The model summary for model II (Table S2) showed a good overall fit, with a slighted higher standard error (+6.08 Ω) and lower *R*^2^ (-1.21%) than model I. The diagnostic tests (Figure S4d–f) showed a normally distributed data with higher residual deviation than in model I. The residual vs run order graph showed that the residuals were mostly randomized. The resistance DoE-RS for model II (Figure 2d) also showed a trend similar to model I, where lower speed and higher power resulted in lower resistance. However, by including the delamination of the material from the substrate (Figure 2e,f), it can be observed that only a narrow region results in a low linear resistance combined with a sturdier LIG. Figure 2 shows the predicted and measured linear resistances for three combinations of speed and power. The best performing region was selected to continue the experiments and was denominated LIG B.

**Figure 3:**
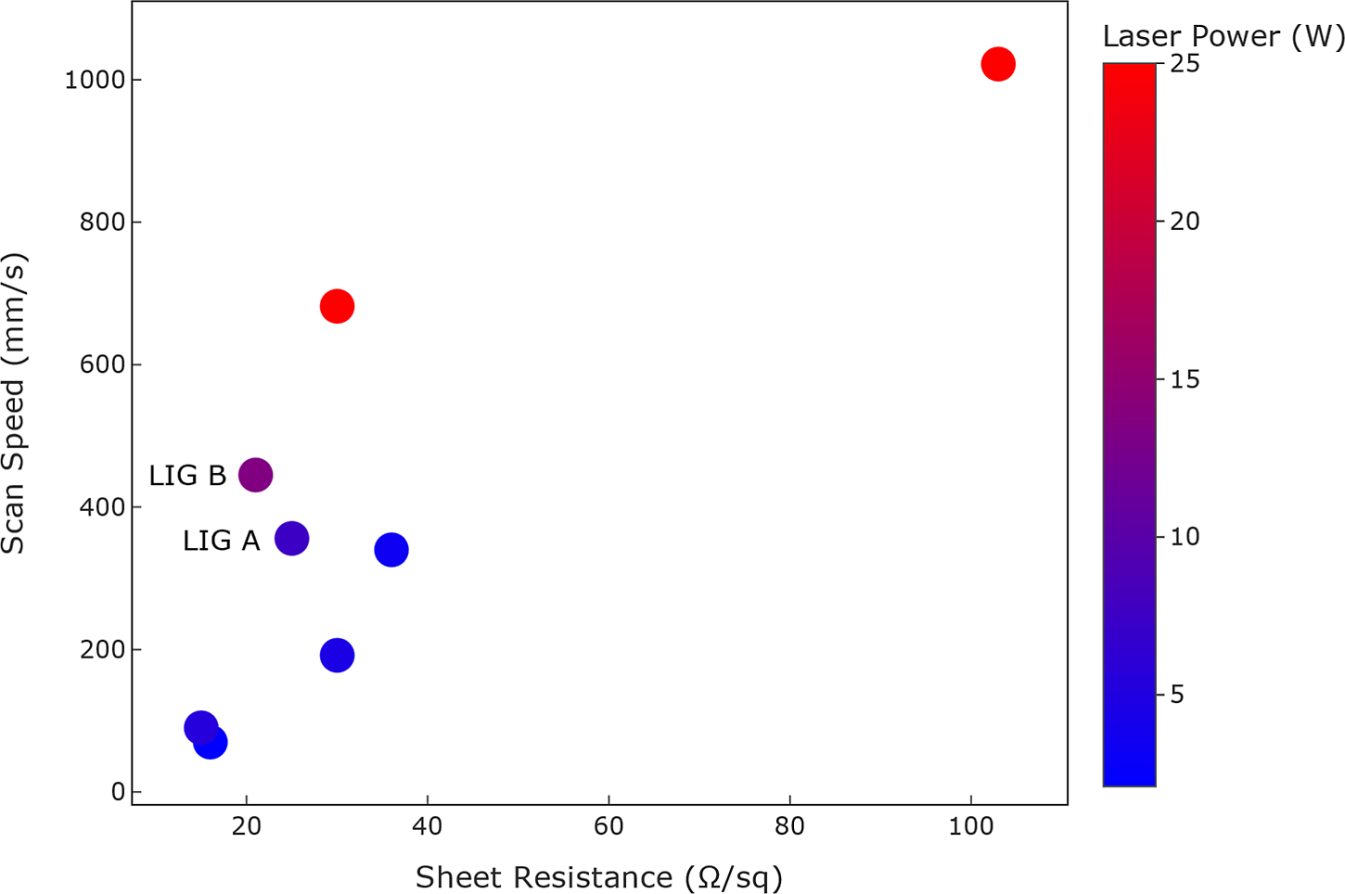
Comparison of sheet resistance values for LIGs fabricated on PI based on laser scan speed and laser power.^8,18,19,38^

To further compare the LIGs, two sheets of LIG A and LIG B that were fabricated on distinctive days had their linear resistance, sheet resistance, and resistivity measured (Table S5). Overall, the results showed that the LIG fabrication was reproducible over different days, and the linear resistance was within the range predicted in Table S3 and showed good correlation with the sheet resistance. The prediction interval was 220–289 Ω for LIG A and 145–253 Ω for LIG B, and from Table S5, the average value for both sheets was 260 ± 10 Ω (*n* = 9) for LIG A and 220 ± 20 Ω (*n* = 9) for LIG B, which are within their respective prediction interval. LIG A and LIG B showed low resistivity values of 750 ± 60 and 630 ± 40 Ω (*n* = 9), respectively. The sheet resistance results were compared to literature values in Figure 3, all using PI as a substrate. The figure demonstrates a relationship among laser power, scan speed, and sheet resistance in LIG fabrication. Notably, low scan speed combined with low laser power generated LIGs with the lowest sheet resistance (15 and 16 Ω/sq).^8,18^ In contrast, higher scan speeds generally correlate with increased sheet resistance, as observed in the LIG fabricated by Cardoso et al., where a scan speed of 1022 mm/s resulted in a sheet resistance of 103 Ω/sq, the highest among all entries.^38^ This suggests that rapid scanning reduces the extent of carbonization, leading to a less conductive material. However, a higher scan speed reduces the time required to fabricate the LIG structure, which is desirable for large-scale applications. LIG A and LIG B, which feature moderate scan speeds of 356 and 445 mm/s, produced sheet resistances of 25 ± 2 and 21 ± 1 Ω/sq, respectively. The sheet resistance is lower than the one found by Murray et al. for a similar scan speed (36 Ω/sq), and the sheet resistance is close to the ones obtained at low scan speed and power.^8,18^ The results indicate that by optimizing laser power and scan speed, low sheet resistance combined with faster production time can be achieved. In the next sections, the LIGs will be compared to the GS-SPE, with further understanding of how the selection of power, speed, and focus affects the carbon composition of the materials.

### 3.2 Material Characterization

**Figure 4:**
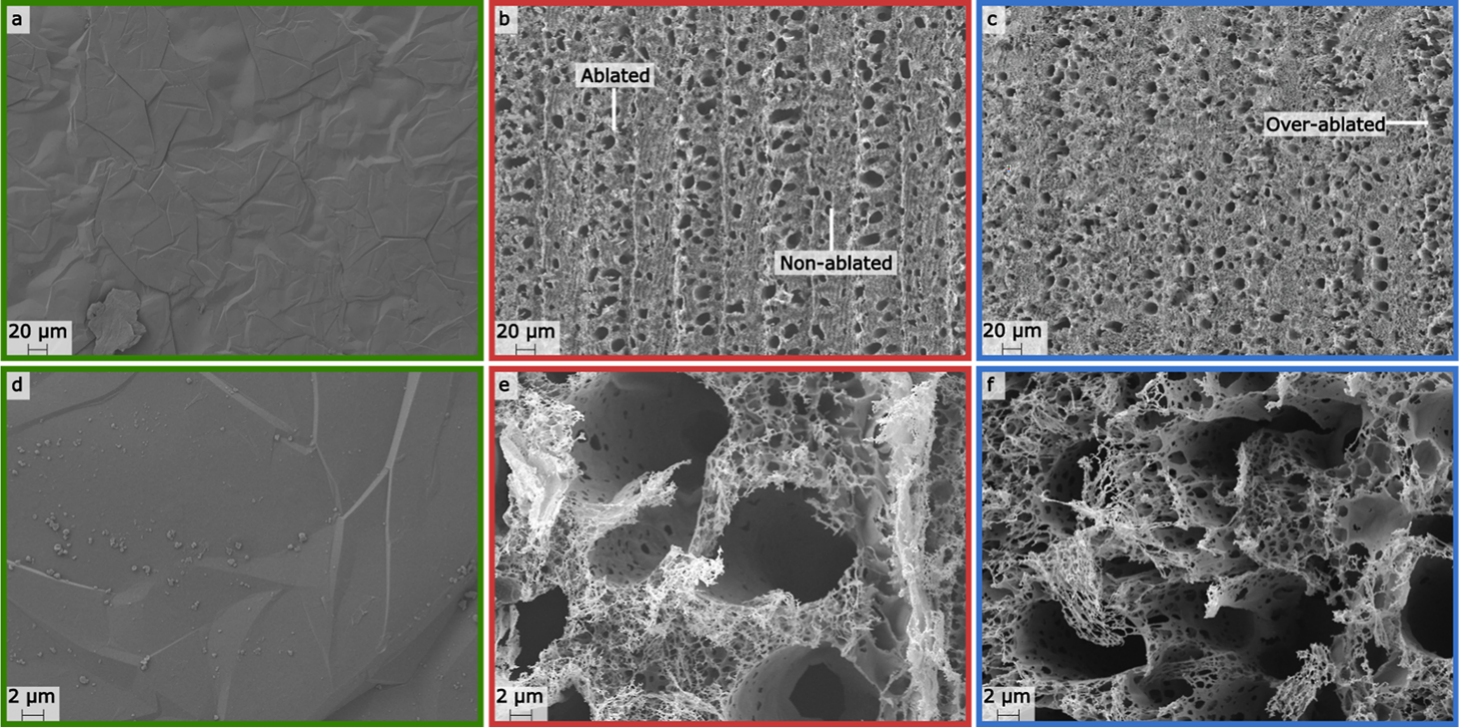
Color-coded SEM images of the GS-SPE (green border, a, d), LIG A (red border, b, e, specifying the ablated and nonablated regions), and LIG B (blue border, c, f, specifying the overablated regions). The top row shows a magnification of 600× and bottom row of 6500×.

An investigation of the LIG morphology was carried out by SEM, Raman, and contact angle analysis and compared to that of the GS-SPE. The surface morphology directly affects the sensing properties of the WE, with geometric surface area, hydrophilicity, and material structure being key properties to be analyzed. Figure 4 shows the two magnification settings of the electrodes. The surface graphene sheet of GS-SPE (Figure 4a,d) is smooth and homogeneous, showing a well-packed layered structure. The elevation difference in the smooth surface could be attributed to the edge of individual sheets or to folded sheet structures.^39^ The smaller fragments on the surface of the graphene sheet (Figure 4d) could be derived either from graphite flakes or the graphene sheet breakage.^40^ Unlike the graphene sheet, the LIG structure is very porous and uneven. The surface of LIG A (Figure 4b,e) contains both larger outer pores and smaller inner pores inside the cylindrical pores (Figures 4e and S5a), similar to what can be seen for LIG B Figures 4c,f and S5b. LIG A shows defined ablated and nonablated regions with an increase in pore numbers in the ablated region (Figure 4b). LIG B has less defined nonablated regions, with some overablated areas occurring probably due to the high average laser power (Figure 4c). Nonablated regions might contribute to the mechanical stability of the carbon film, as LIGs are usually brittle in nature.^41^

**Figure 5:**
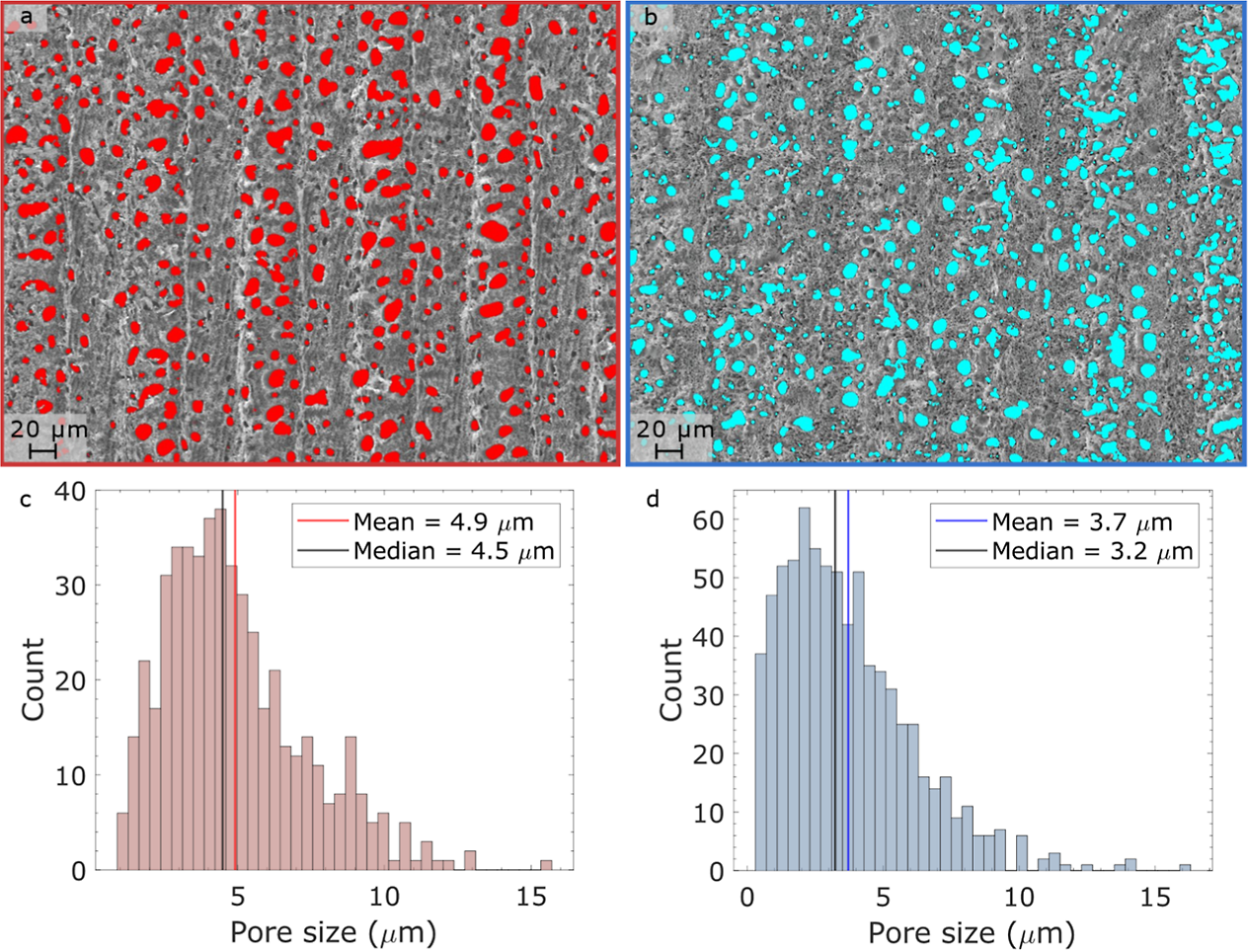
Color-coded figures corresponding to LIG A (red) and LIG B (blue). (a,b) Colored distribution of pores on the surface of the LIGs. (c,d) Pore size distribution considering the pore radius.

To further investigate the porosity of the LIGs, the pore distribution was processed and analyzed (Figure 5a,b). It was more difficult to distinguish the pore distribution on LIG B than on LIG A due to the shape and depth of the pores. The distribution of the pore sizes can be seen in Figure 5c,d, with LIG A presenting a total of 503 pores and LIG B 754. Although LIG B had more pores, the size was overall smaller, with an average radius of 3.7 μm versus that of 4.9 μm for LIG A. LIG A was fabricated using a 7.5 W average laser power, while LIG B used 13.5 W. As previously shown, the LIG porosity increases with average laser power.^8^ In this case, however, the total area covered by the pores was 13% for both LIGs, indicating that the higher speed used for LIG B might have counterbalanced the average laser power effect. Another interesting aspect of the LIGs was the presence of smaller nanopores inside larger pores (Figure 4e,f). The difference in the final structure of the pores is clear, with granular nanoparticles forming on LIG A, while LIG B produced a thinner layer. This artifact occurs probably because of the lower engraving speed of LIG A.^41^ The cross-sectional SEM images (Figure S6) revealed that both LIGs have similar thicknesses, with an average thickness of 53 ± 7 μm. The graphene sheet on the GS-SPE had a thickness ranging from 10 to 70 μm; however, this seems to be because it started to peel off from the carbon paste at certain parts. From the magnified cross-section images, it can be observed that LIG B (Figure S5a) has many small pores in a honeycomb-like structure, while LIG A (Figure S5b) has larger cylindrical pores.

**Figure 6:**
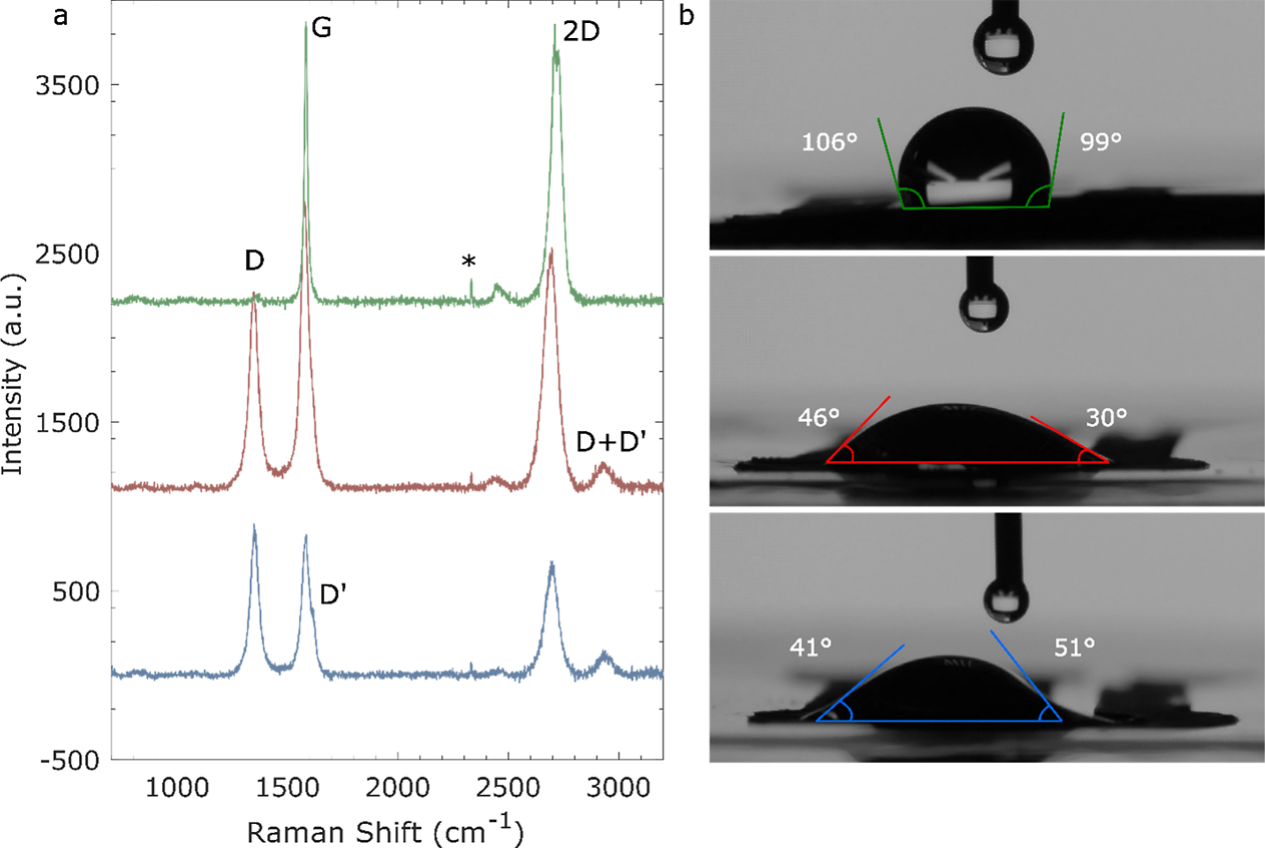
(a) Raman spectra of the GS-SPE (green), LIG A (red), and LIG B (blue). The D, G, D′, and 2D bands are highlighted in the graph. (b) Contact angle analysis of the GS-SPE (green), LIG A (red), and LIG B (blue).

According to previous studies, the defect density on graphene materials impacts the electrochemical activity of the chemical sensors. For example, graphene WEs with disordered materials and more defects can increase the electrochemical performance of the device.^42^ Raman spectroscopy was conducted to differentiate the carbon composition and crystal order of the fabricated electrodes (Figure 6a). The peak marked with an asterisk (*) at around 2330 cm^–1^ can be attributed to the presence of N_2_. This peak can appear due to the interaction of the laser with N_2_ molecules present in the environment or within the sample chamber.^43^ The G and 2D peaks were present in all electrodes, while the LIGs also presented the D peak. The D and D′ band represents defects in the crystal lattice due to breakage of the sigma bonds.^25^ The D band was present at 1347 cm^–1^ in both LIG A and LIG B Raman spectra and could also be detected on the GS-SPE spectrum, although at much lesser intensity. The D′ could be faintly observed for LIG B but did not seem to be present in LIG A nor in the GS-SPE spectrum. The G and 2D bands are related to highly ordered graphitic structures. The 2D band correlates to the formation of graphene layers, deriving from the stacking of graphene layers on the *c*-axis.^8^ A minor band around 2930 cm^–1^ was observed in both LIG A and LIG B, but it was not observed in the GS-SPE spectrum. This corresponds to the D + D′ band, which indicates the disorder structure of graphene with oxygen containing groups.^44^ From the Raman spectra, it can be concluded that although LIG A is visually similar to LIG B (Figure 6), they have slightly different carbon structures. LIG A is between the highly ordered GS-SPE and the disordered LIG B, while LIG B presents the most defects.

The Raman peak parameters and the *I*_D_/*I*_G_ and *I*_2D_/*I*_G_ peak area ratio were calculated for all electrodes (see Table S6). The ratio *I*_D_/*I*_G_ can be related to the degree of disorder of the material. GS-SPE, LIG A, and LIG B presented *I*_D_/*I*_G_ ratios of 0.03, 0.68, and 0.97, respectively, indicating increasing levels of structural disorder from GS-SPE to LIG B and *I*_2D_/*I*_G_ ratios of 3.30, 1.37, and 1.30, respectively, indicating decreasing number of graphene layers or lower quality monolayer graphene from GS-SPE to LIG B, which affects electron mobility. The high *I*_D_/*I*_G_ ratio of LIG B (0.97) suggests that it has numerous defects, which is advantageous for electrochemical sensing due to the increased number of active sites available for the electrochemical reactions involved in nitrite detection. This can lead to enhanced sensitivity and lower detection limits for nitrite ions.^42^ However, it also presents the lowest *I*_2D_/*I*_G_ ratio of 1.30 and likely has the lowest electron mobility among the three electrodes. LIG A, with a lower *I*_D_/*I*_G_ ratio of 0.68 and slightly higher *I*_2D_/*I*_G_ ratio than LIG B, likely has fewer defects and a more ordered structure, which may result in a relatively lower sensitivity but can still be effective for nitrite sensing applications. Lastly, with the highest *I*_2D_/*I*_G_ ratio of 3.30, GS-SPE has the highest quality monolayer graphene among the electrodes, as confirmed in Figure 4a,d. This high-quality graphene ensures excellent electron mobility, but the low *I*_D_/*I*_G_ ratio of 0.03 suggests minimal defects, potentially resulting in fewer active sites for nitrite detection. Therefore, while GS-SPE might be very stable and reproducible, its sensitivity for nitrite sensing could be lower compared to those of more defective materials.

To better understand the hydrophilic/hydrophobic behavior of the electrodes, the surface wettability was studied using the contact angle analysis (Figure 6b). The measurements were repeated three times for each electrode type. Measuring the surface wettability is important because it can affect the sensing properties of the WE. For example, in ion-selective electrodes, a hydrophobic WE is desired as it prevents the formation of a water layer between the ion-selective membrane and the WE conductive material, while in this case, since the WE is not treated and is directly oxidizing the analyte, a hydrophilic behavior is more advantageous.^25,45^ The GS-SPE has a more hydrophobic surface, with an average contact angle of θ = 87° ± 17°. This is likely due to its defect-free, homogeneous surface, which probably decreases the number of oxygen atoms on its surface. On the other hand, both LIGs presented a more hydrophilic surface with θ = 35° ± 10° for LIG A and θ = 38° ± 9° for LIG B. This is probably a combination of their high porosity with the presence of heteroatoms on their surface, such as oxygen.^25^

### 3.3 Electrochemical Analysis

**Figure 7:**
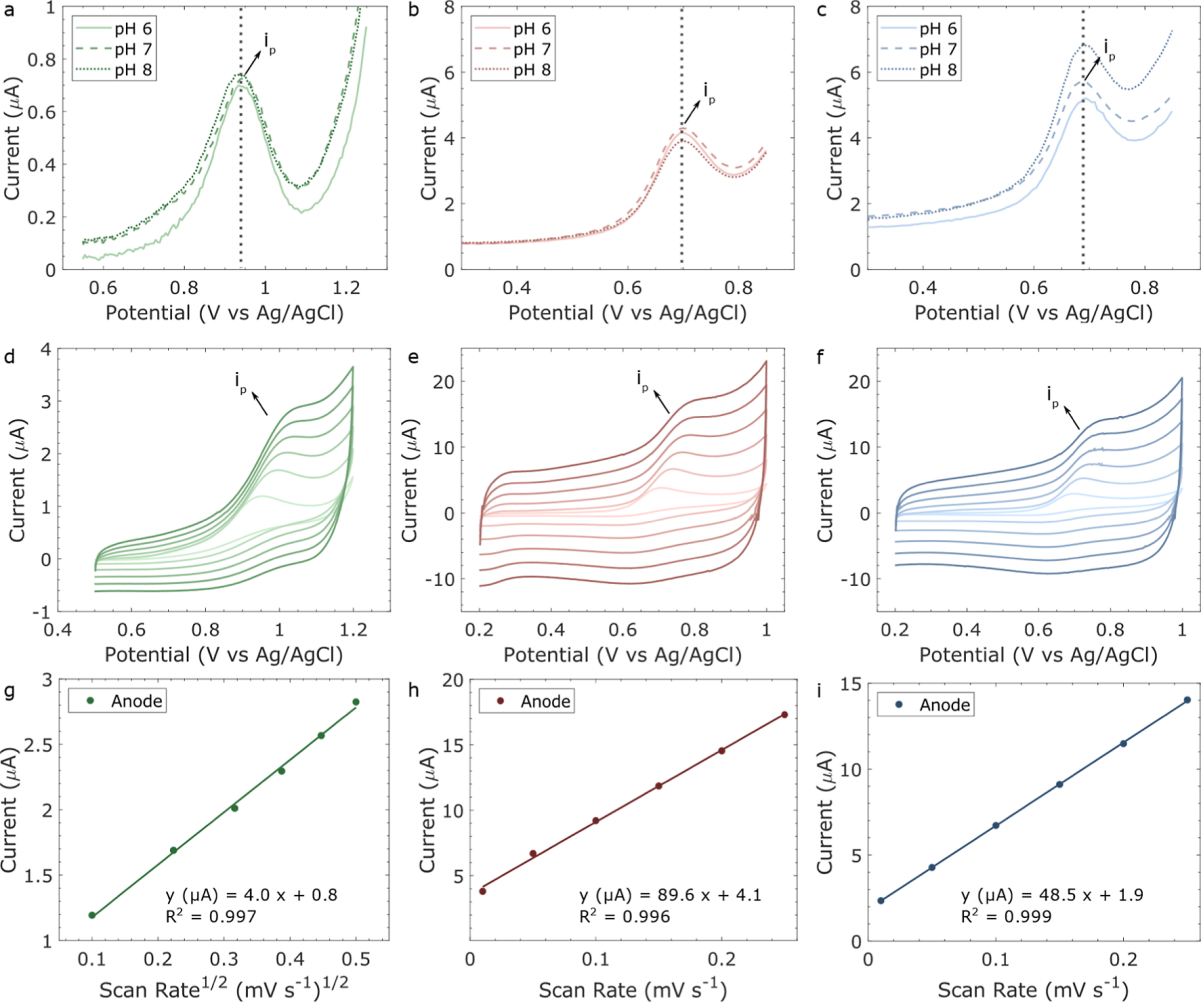
50 μM NaNO_2_ DPV at pH 6, 7, and 8 with 50 mM NaCl using the (a) GS-SPE, (b) LIG A, and (c) LIG B. CV scan rate of 10 to 250 mV/s for 100 μM NaNO_2_ in 50 mM NaCl pH 7 for the (d) GS-SPE, (e) LIG A, and (f) LIG B. The CV scan rate linear fit of the peak current vs (g) square root of the scan rate for the GS-SPE, (h) scan rate for LIG A, and (i) scan rate for LIG B. The *i*_p_ current corresponds to the oxidation peak between +0.8 and +1.0 V for the GS-SPE and +0.6 and +0.8 V for the LIGs.

Further analysis was performed for all electrodes to investigate how the material composition and surface morphology affected the electrochemical performance. For this, the oxidation of NO_2_^–^ was chosen, as it allows the use of the bare carbon WE to be employed, as carbon materials have been shown to oxidize NO_2_^–^ into NO_3_^–^ at positive potentials.^33^ First, the sensors were tested at pH 6, 7, and 8 in 50 mM NaCl aqueous solution with 50 μM NO_2_^–^ to understand if a change in pH affects the current intensity of the NO_2_^–^ oxidation peak. Although acidic pH has been shown to be optimum for the detection of NO_2_^–^ when using LIG sensors due to the presence of electrostatic repulsion at higher pH, the pH range of 6–8 was chosen as it is representative of the drinking water pH (6.5 to 9.5 according to the EU directives).^31,36,46^ The DPV can be observed in Figure 7a–c, and the difference in peak current (Δ*i*_p_) per pH is listed in Table S7. A few observations can be made from this analysis. First is that the LIG B seems to produce a higher peak current (*i*_p_) than the other sensors, while also having the largest variation in Δ*i*_p_. LIG A had similar Δ*i*_p_ to the GS-SPE; however, it also presented a much higher *i*_p_ overall. The higher oxidation current observed for the LIGs is probably due to the higher geometric surface area of the electrodes alongside a more hydrophilic surface, as observed in the SEM images (Figures 4 and S5) and the contact angle images (Figure 6b). This allows for a better interaction between the analyte and highly porous surface of the LIGs, which possibly increases the electroactive area of the WE. Future studies on the electroactive area of the electrodes could provide additional evidence that the increased geometric surface area observed in the LIGs’ SEM images is also electrochemically active, thus contributing to the higher sensitivity. For the rest of the electrochemical experiments, pH 7 was used, as it corresponds to a neutral pH.

The effect of the scan rate (10 to 250 mV/s) on the NO_2_^–^ oxidation reaction was investigated, as shown in Figure 7d–i. The reaction is irreversible, and *i*_p_ increases with the increase of the scan rate for all electrodes; however, the governing reaction mechanism is different for the GS-SPE and the LIGs. For the GS-SPE, the *i*_p_ is linearly proportional to the square root of the scan rate (Figure 7g), indicating that the oxidation of nitrite is a diffusion-controlled process.^36^ For the LIGs, however, *i*_p_ is linearly proportional to the scan rate (Figure 7h,i). This could indicate that the reaction is mass-transfer limited in this case due to, for example, adsorption of the ions on the electrode surface.^47^

EIS was also performed to study the surface properties of the sensors. Figure S7 shows the different Nyquist plots for each sensor over a range of NO_2_^–^, from 10 to 500 μM. GS-SPE (Figure S7a,d) clearly shows a behavior different from LIG A (Figure S7b,e) and LIG B (Figure S7c,f). For the GS-SPE, there is a decrease in imaginary resistance with increasing concentration. As the concentration of the electrolyte increases, the ionic strength increases, leading to a more compact double layer. This results in enhanced charge transfer kinetics and a reduced diffusion impedance.^48,49^ This is in agreement with the results from Figure 7d,g, which indicates a diffusion-controlled process for the oxidation of nitrite for the GS-SPE. For LIG A and LIG B, the double-layer capacitance region remains almost constant across varying concentrations of the electrolyte, indicating that the surface properties of the electrode, such as surface area and surface roughness, are more dominant than the bulk properties of the electrolyte.^49^ This can be correlated to the porous surface of both LIGs as seen in the SEM images (Figure 4) and the fact that the analyte oxidation is a surface-controlled process (Figure 7). There is also a large difference in the imaginary resistance at low frequencies between the GS-SPE and the LIGs of about 20 to 40 times when no NO_2_^–^ is present in the solution to similar resistances at 500 μM. While LIG A and LIG B had similar EIS responses, LIG B had lower real and imaginary impedances, which could reflect in faster charge transfer and better conductivity for nitrite sensing and explain the higher currents observed in Figure 7c.

**Figure 8:**
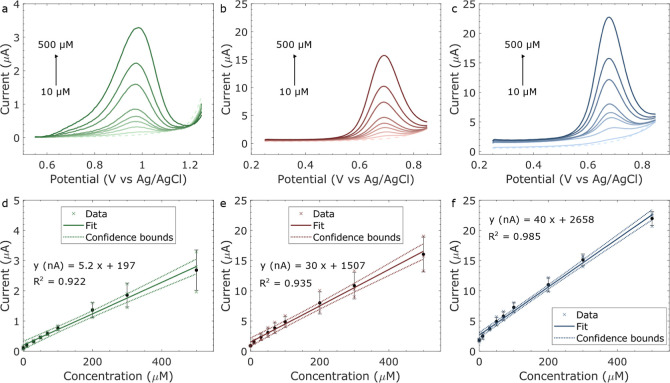
DPV of 10 to 500 μM of NaNO_2_ in 50 mM NaCl pH 7: (a) GS-SPE, (b) LIG A, and (c) LIG B. The baseline is represented by dotted lines. The calibration plot inset for (d) GS-SPE, (e) LIG A, and (f) LIG B.

DPV was employed to investigate the faradaic response of the sensors toward NO_2_^–^, to minimize the capacitive charging of the LIGs that appears in techniques such as the CV. This, in turn, improves the NO_2_^–^ oxidation signal, making it possible to analyze even lower concentrations of the analyte. The DPV response of the sensors from 10 to 500 μM NO_2_^–^ and the calibration plot can be observed in Figure 8. It can be noticed that the oxidation potential is higher for the GS-SPE than for the LIGs. This suggests that the material composition of the LIGs is more efficient at oxidizing the NO_2_^–^ ion as it requires less input energy. This peak shift, which is also observed in Figure 7, could be explained by the transition from pristine graphene to LIG. LIG has a higher defect density and a porous structure, providing more electroactive sites and enhancing electron transfer during electrochemical reactions. These defects lower the activation energy for redox processes, resulting in shifts in peak potential. This phenomenon has been documented in other graphene modified materials, where the introduction of defects or functional groups typically shifts peak potentials due to altered electron transport properties and local chemical environments.^50,51^ Similar to the previous electrochemical results, GS-SPE had the lowest oxidation current of all electrodes. On average, LIG A had a *i*_p_ of 565% higher than the GS-SPE and LIG B, of 729%. The higher electrochemical activity of LIG B is likely correlated to its higher disordered structured (Figure 6a and Table S6), as defects and disordered material structure can increase the electrochemical performance of the device.^42^ The DPV peaks for GS-SPE are also broader than the LIG ones. This could be attributed to a diffusion-controlled process versus a surface-controlled process as discussed in the CV scan rates (Figure 7) and EIS analysis (Figure S7).

Sensor performance was evaluated by measuring the sensitivity, linearity, relative standard deviation (RSD), and limit of detection (LOD). All electrodes presented an increase of *i*_p_ with an increase of analyte, presenting a linear correlation with *R*^2^ greater than 0.9 throughout the whole NO_2_^–^ concentration range. The summary of the calibration plots can be found in Table S8. The RSD was calculated by taking the SD of each concentration, dividing it by its corresponding average *i*_p_, and multiplying it by 100 to obtain a percentage, with RSD = 100*SD [concentration]/*i*_p_ [concentration]. LIG B presented the best linearity with a RSD <21% and a sensitivity of 40 ± 1 nAμM^–1^. LIG A followed closely behind with a RSD <25% and a sensitivity of 30 ± 2 nAμM^–1^. There was a difference of 10 nAμM^–1^ in sensitivity between both LIGs, and the GS-SPE displayed the smallest sensitivity of the three, at only 5.2 ± 0.3 nAμM^–1^. LOD was estimated using the IUPAC definition of LOD = 3.3*σ/s, where σ is the SD of the background current and *s* is the sensitivity of the calibration plot.^52^ The LOD was higher than the lowest tested concentration (10 μM) for both GS-SPE (LOD of 37 μM) and LIG B (LOD of 19 μM), while LIG A had an LOD slightly lower than 10 μM (9 μM). This could be improved by defining two linear ranges for the sensor, for example, one fitting for the lower concentration range and another for higher concentrations, as the slope changes slightly from medium to higher concentrations. For this, further points between the studied concentrations should be added for more accurate results.

In comparison to other printed NO_2_^–^ sensors found in the literature, the LIGs developed here presented a good performance (Table 2). It is especially interesting that the high sensitivity was achieved without the addition of nanoparticles or other carbon additives, as with other NO_2_^–^ LIG sensors.^36,41^ By optimizing the laser parameters using the DOE, incredibly sensitive binder-free NO_2_^–^ sensors were achieved in a single-step process. Furthermore, the fabricated sensors appear to function similarly at different pH values (Figure 7), making them ideal for quick routine samples, either in the field or in the laboratory.

**Table 2:** Various Parameters of Printed Sensors for the Detection of NO_2_^–^a

printing method	sensing material	detection method	linear range (μM)	LOD (μM)	sensitivity (nAμM^–1^ cm^–2^)	ref
screen-printing	Au NPs/GOx	DPV	1–6000	0.13	305	(53)
laser engraving	chitosan	DPV	2–1000	0.9	121	(41)
laser engraving	Au NPs/CNTs	SWV	10–140	0.9	183	(36)
laser engraving	bare LIG	DPV	10–70	0.27	585	(44)
laser engraving	bare LIG	DPV	10–500	9	420	this work
laser engraving	bare LIG	DPV	20–500	19	570	thiswork
screen-printing	graphene sheet	DPV	38–500	37	73	this work

a CNT = Carbon nanotube; GOx: Graphene oxide; NPs = nanoparticles; SWV = Square wave voltammetry.

**Figure 9:**
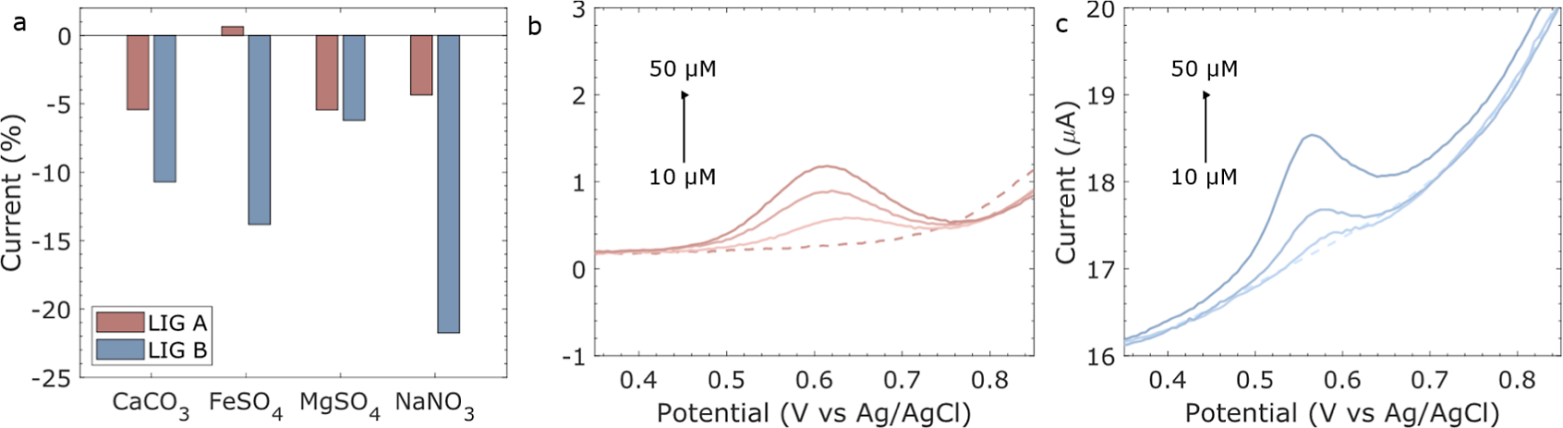
(a) Peak current difference of 50 μM NaNO_2_ in the presence of 50 μM of each common interfering ion (CaCO_3_, FeSO_4_, MgSO_4_, or NaNO_3_) for LIG A and LIG B. DPV of 10 to 50 μM NaNO_2_ in spring water for the: (b) LIG A and (c) LIG B. The baseline is represented by dotted lines.

To investigate the selectivity of these sensors in the field, the *i*_p_ signal of 50 μM of the LIGs was compared in the presence of common ionic compounds (CaCO_3_, FeSO_4_, MgSO_4_, and NaNO_3_) present in drinking and river water (Figure 9a). From Figure 9a, it can be seen that the *i*_p_ of LIG B is highly influenced by the addition of other analytes, specially NaNO_3_. On the other hand, the NO_2_^–^ oxidation signal is more stable for LIG A even in the presence of other ions. This could be due to the different carbon structure of LIG A compared to LIG B; however, further analysis is needed to support this. To test the sensors’ performance in drinking water, a bottled spring water was used without any additional modification. The added NO_2_^–^ concentration was in the range of 10–50 μM. Although the same trend of current increase with analyte increase was observed for both electrodes, the *i*_p_ found was on average half of that found in the previous DPV studies (Figure 8b,c) for LIG A and a third for LIG B. This could be because of the interference of other ions present in the drinking water, which decreased the *i*_p_. This indicates that the calibration curve for the LIGs needs to be adjusted, depending on the medium being used.

## 4 Conclusions

The current work demonstrated that LIGs are a suitable replacement for carbon-based SPEs as electrochemical sensors, as they offer the advantages of a graphene-like structure without the need to use binders or solvents. To achieve this, the linear resistance and the carbon adhesion of the LIGs to the PI substrate were improved by using the DoE-RS statistical approach. The instrument parameters that were used to construct the DoE-RS models were the average laser power, speed, and focus, as they seem to influence the quality of the LIGs the most. The linear resistance DoE-RS models had a *R*^2^ > 95%; however, only when combined with the delamination models could suitable LIGs be fabricated. That is because depending on the laser parameters used the LIGs become brittle, even though they had a lower resistance. From the models, two regions with low resistance and good substrate adherence were chosen to fabricate the LIGs, resulting in LIG A (25 ± 2 Ω/sq) and LIG B (21 ± 1 Ω/sq). Detailed morphological and electrochemical characterization were performed and the LIGs were then compared to the GS-SPE.

The SEM images showed that the LIGs were highly porous, having a geometric surface area higher than that of the continuous graphene. From the Raman spectroscopy, it was possible to infer that the LIGs showed a disordered graphene structure, which has been shown to positively influence electrochemical activity, while the GS-SPE was constituted of ordered graphene layers. The fabricated LIGs were also more hydrophilic than the GS-SPE, which is advantageous when using pristine WE for electrochemical detection in water. These facts combined can increase the electrochemical performance of the device, which was demonstrated by the electrochemical studies on the oxidation of NO_2_^–^ ions. The LIGs were overall more stable and presented a peak current higher than 500% when compared to that of the GS-SPE. Furthermore, these binder-free, unmodified LIGs exhibited sensitivity comparable to other modified SPEs or LIGs for NO_2_^–^ detection. Although NO_2_^–^ was chosen as the analyte of interest, the LIGs fabricated here can be used as templates to detect other analytes by further modifying the WE with other materials. Overall, the potential of using DoE-RS as a resource-efficient fabrication tool to optimize LIG electrochemical sensors was demonstrated. Further optimization through another round of DoE-RS could be achieved by combining the response variables used in this study (resistance and delamination) with other outputs such as such as Raman spectra and/or electrode kinetics. Further studies are also needed to fully assess these LIGs potential, such as long-term surface degradation and stability studies, and how they compare to other state-of-the-art sensors.

## Data Availability

The data supporting the findings reported in this paper are available under a CC BY 4.0 licence from the Enlighten: Research Data repository at https://doi.org/10.5525/gla.researchdata.1794.

## Supporting Information Available

Details of the DOE analysis, including the analysis summary and prediction models; SEM of the inner pores of the LIGs; cross-sectional SEM; summary of Raman spectra peaks; EIS analysis; and calibration curve parameters (PDF)
