# Supplementary material for: Optimizing Carbon Structures in Laser-Induced Graphene Electrodes Using Design of Experiments for Enhanced Electrochemical Sensing Characteristics
Source: ACS Appl Mater Interfaces. 2024 Nov 14;16(47):65489–502. doi: 10.1021/acsami.4c13124 (PMC11615855; doi:10.1021/acsami.4c13124)
Supplement: Supplementary file 1 — am4c13124_si_001.pdf [file am4c13124_si_001.pdf]

Supporting Information

Optimising Carbon Structures in Laser-Induced Graphene Electrodes Using Design of Experiments for Enhanced Electrochemical Sensing Characteristics

Fabiane Fantinelli Franco[a*], Muhammad Hassan Malik[b]. Libu Manjakkal[c], Ali Roshanghias[b], Cindy J. Smith[a], Caroline Gauchotte-Lindsay[a*]

[a]Water and Environment Group, Infrastructure and Environment Division, James Watt School of Engineering, University of Glasgow, Glasgow, G12 8LT, UK.
[b] Silicon Austria Labs GmbH, Europastrasse 12, A-9524 Villach, Austria.
[c] School of Computing and Engineering & the Built Environment, Edinburgh Napier University, Merchiston Campus, EH10 5DT, UK

*Corresponding authors: fabifantinelli@gmail.com; caroline.gauchotte-lindsay@glasgow.ac.uk

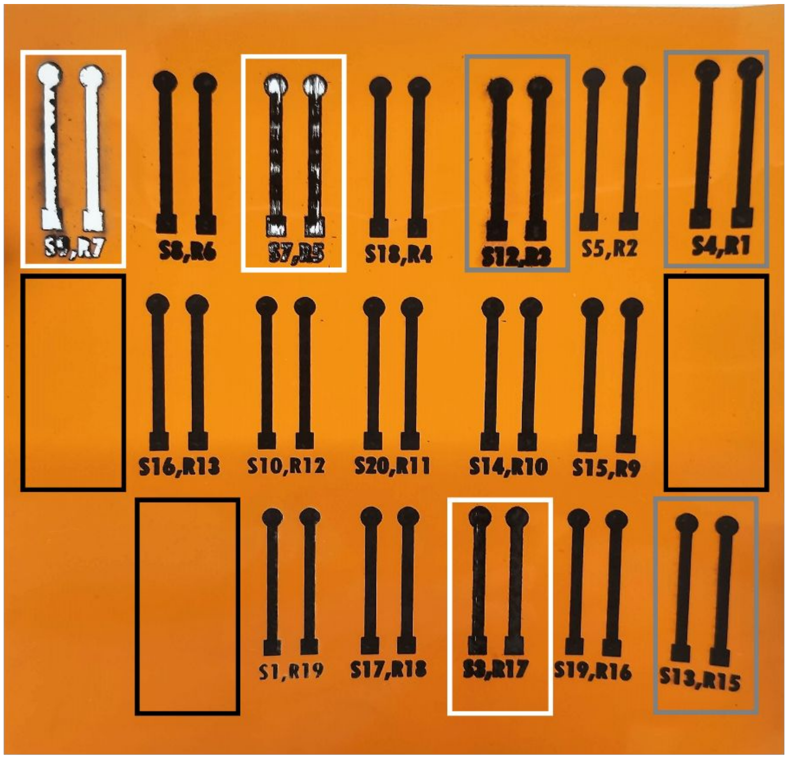

Sample No.	Run No.	Speed (%)	Power (%)	Focus (mm)	Resistance (Ω)
4	1	30	20	0.5	501
5	2	10	5	1.5	507
12	3	20	25.11	1	323.5
18	4	20	12.5	1	500.5
7	5	10	20	1.5	403
8	6	30	20	1.5	643
9	7	3	12.5	1	10000
11	8	20	-0.11	1	10000
15	9	20	12.5	1	496.5
14	10	20	12.5	1.84	438
20	11	20	12.5	1	496
10	12	37	12.5	1	994
16	13	20	12.5	1	492.5
2	14	30	5	0.5	10000
13	15	20	12.5	0.16	417
19	16	20	12.5	1	494.5
3	17	10	20	0.5	135.5
17	18	20	12.5	1	494
1	19	10	5	0.5	374
6	20	30	5	1.5	10000

Figure S1. Photo of the 3-factor (speed, power, and focus) DoE (top). The label corresponds to the sample number and the DoE run number (e.g., sample 4, run 1 corresponds to S4R1). The black rectangles represent the areas where no graphitisation of the substrate occurred; the white rectangles where the substrate was partially/fully cut; the grey rectangles where a more fibrous LIG was produced. Table showing the parameters used for each combination of sample and run number (down). A power of 5 to 20% corresponds to 3.75 to 15 W. Where no graphitisation occurred, a high resistance of 10 kΩ was added to run the DoE-RS.

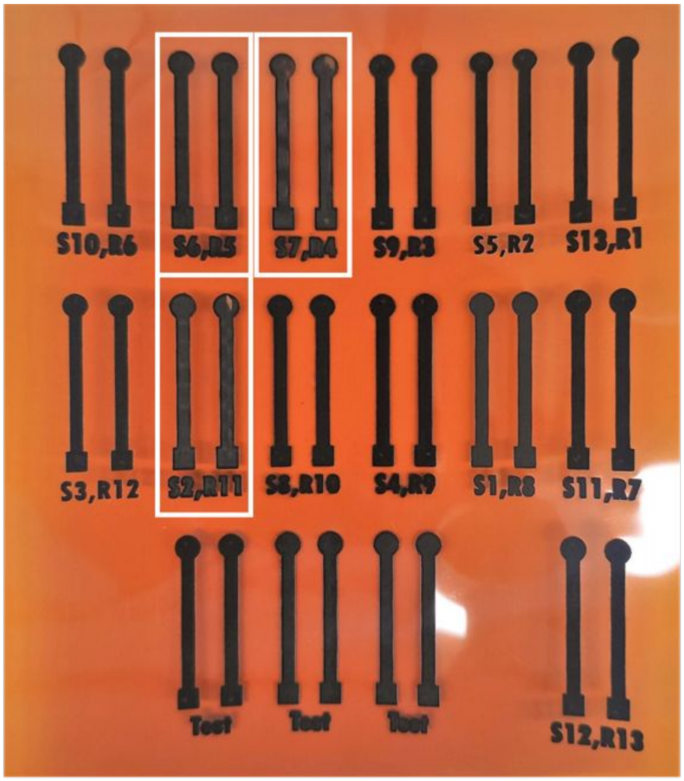

Sample No.	Run No.	Power (%)	Speed (%)	Resistance (Ω)	Delamination
13	1	15	15	226.5	0
5	2	7.9	15	228	0
9	3	15	15	226.5	0
7	4	15	8	126	1
6	5	22	15	157	1
10	6	15	15	214.5	0
11	7	15	15	228	0
1	8	10	10	172.5	0
4	9	20	20	280	0
8	10	15	22	289.5	0
2	11	20	10	102	1
3	12	10	20	245	0
12	13	15	15	218.5	0

Figure S2. Photo of the 2-factor (speed, power) DoE before bending (top). The label corresponds to the sample number and the DoE run number (e.g., sample 13, run 1 corresponds to S13R1). The white rectangles represent the areas where delamination partially or fully occurred after bending and was then set to 1 on the DoE-RS. Table showing the parameters used for each combination of sample and run number (down). A power of 10 to 20% corresponds to 7 to 15 W.

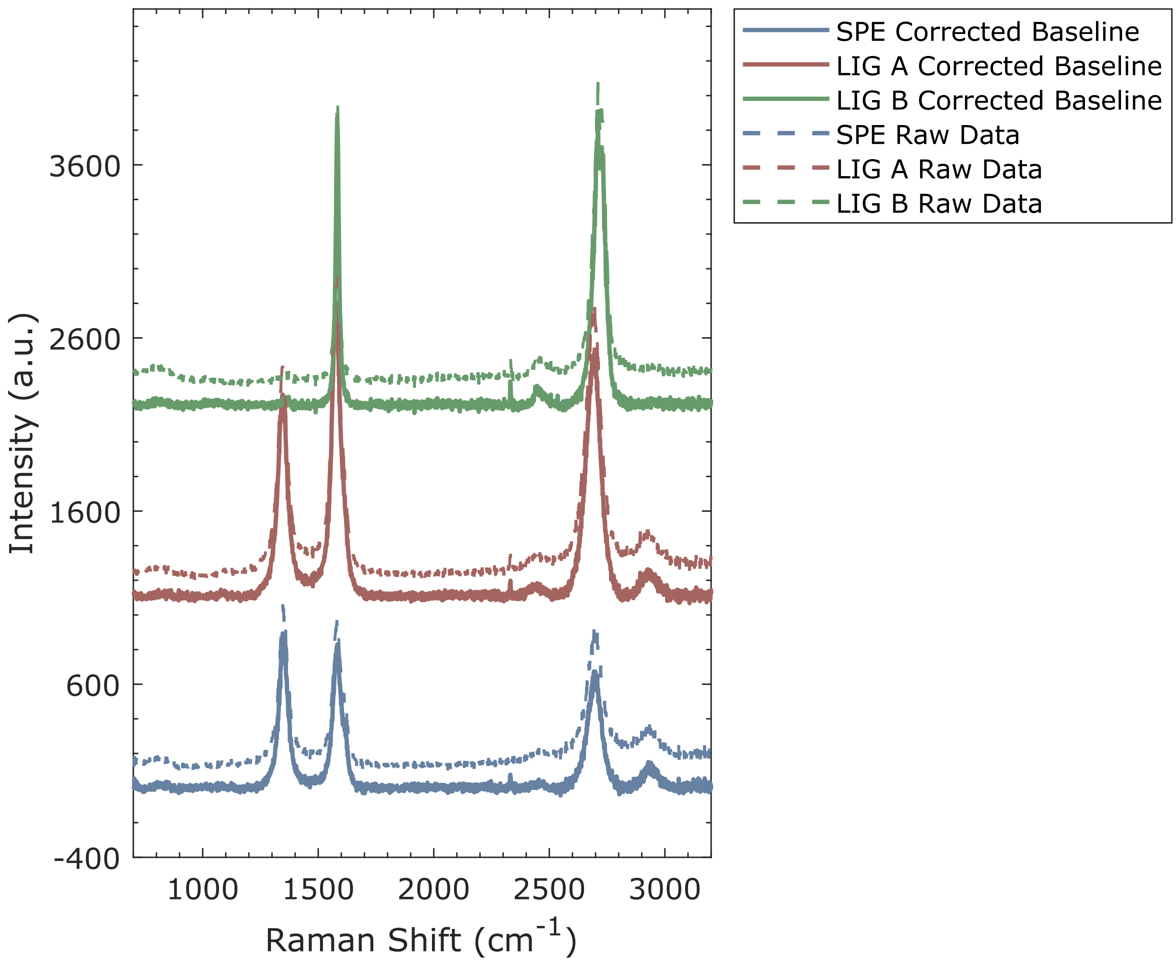

Figure S3. Raw (dashed line) and subtracted baseline (solid line) Raman spectra of the GS-SPE (green), LIG A (red), and LIG B (blue).

Table S1. Analysis of variance for model I and the resistance equation based on the best-fitting model.

Analysis of Variance					
**Source**	**DF**	**Adj SS**	**Adj MS**	**F-Value**	**p-Value**
**Model**	4	35523.8	8881	63.32	0
**Linear**	2	31364.4	15682.2	111.82	0
**Power (%)**	1	2346.1	2346.1	16.73	0.003
**Speed (%)**	1	29018.2	29018.2	206.9	0
**Square**	1	1428.5	1428.5	10.19	0.013
**Power (%)*Power (%)**	1	1428.5	1428.5	10.19	0.013
**Speed (%)*Speed (%)**	1	2782.6	2782.6	19.84	0.002
**2-Way Interaction**	1	2782.6	2782.6	19.84	0.002
**Power (%)*Speed (%)**	8	1122	140.3		
**Error**	4	980.2	245.1	6.91	0.044
**Lack-of-Fit**	4	141.8	35.5		
**Pure Error**	12	36645.8			
**Total**	196.3 - 2.13 Power - 3.72 Speed - 0.571 Power*Power + 1.055 Power*Speed				

DF = degrees of freedom; SS = sums of squares; MS = mean square.

Table S2. Model summary of model I and model II.

	Standard Error (Ω)	R-squared	R-squared (adjusted)	R-squared (predicted)
**Model I**	11.84	96.94%	95.41%	86.80%
**Model II**	17.92	95.73%	92.68%	75.89%

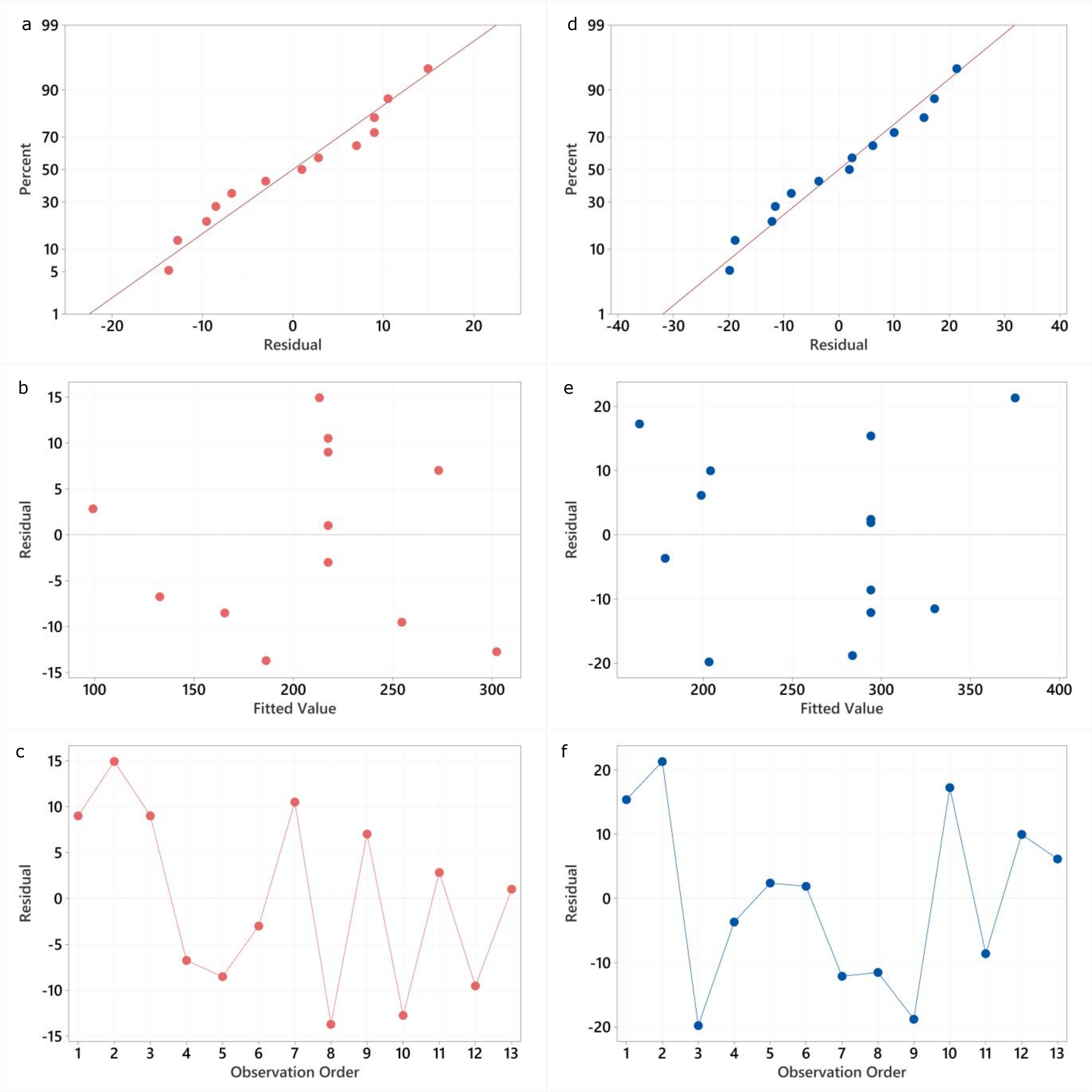

Figure S4. Diagnostic tests for the validation of the ANOVA test for the model I (left column) and model II (right column). a and d) Normal % probability against externally studentised residuals. b and e) Externally studentised residuals against predicted values. c and f) Externally studentised residuals against run order.

Table S3. Predicted and measured resistance based on model I and model II. Highlighted values correspond to LIG A and LIG B.

	Average Laser Power (%)	Speed (%)	Fit (Ω)	SE (Ω)	95% CI (Ω)	95% PI (Ω)	Measured (Ω)
**Model I**	**10**	**20**	**255**	**9**	**233 - 276**	**220 - 289**	**245**[1]
	15	12.5	187	5	176 - 198	158 - 217	148
	17.5	20	279	7	264 - 294	248 - 310	283
**Model II**	**17.9**	**25**	**199**	**14**	**166 – 232**	**145 – 253**	**205**[2]
	20	22	222	11	196 – 247	172 – 271	242
	25	27	318	8	300 - 336	272 – 365	300

SE = Standard error; CI = confidence interval; PI = prediction interval.
1 – LIG A settings
2 – LIG B settings

Table S4. Analysis of variance for model II the resistance equation based on the best-fitting model.

Analysis of Variance					
**Source**	**DF**	**Adj SS**	**Adj MS**	**F-Value**	**p-Value**
**Model**	5	50362.5	10072.5	31.37	0
**Linear**	2	29869.1	14934.5	46.51	0
**Power (%)**	1	21.8	21.8	0.07	0.802
**Speed (%)**	1	29847.3	29847.3	92.95	0
**Square**	2	18504	9252	28.81	0
**Power (%)*Power (%)**	1	18284.9	18284.9	56.94	0
**Speed (%)*Speed (%)**	1	41.4	41.4	0.13	0.73
**2-Way Interaction**	1	1849	1849	5.76	0.047
**Power (%)*Speed (%)**	1	1849	1849	5.76	0.047
**Error**	7	2247.8	321.1		
**Lack-of-Fit**	3	1781.1	593.7	5.09	0.075
**Pure Error**	4	466.7	116.7		
**Total**	12	52610.3			
**Resistance (Ω)**	-698 + 71.0 Power (%) - 4.2 Speed (%) - 1.844 Power (%)*Power (%) - 0.100 Speed (%)*Speed (%) + 0.860 Power (%)*Speed (%)				

Table S5. Linear resistance, sheet resistance, and resistivity for LIG A and LIG B fabricated on different days.

		**Linear Resistance (Ω)**	**Sheet Resistance (Ω /sq)**	**Resistivity (µΩ·m)**
**LIG A**	Sheet I (n=3)	270 ± 10	27 ± 2	820 ± 60
	Sheet II (n=6)	255 ± 6	23.5 ± 0.3	710 ± 20
	**Average (n=9)**	**260 ± 10**	**25 ± 2**	**750 ± 60**
**LIG B**	Sheet I (n=3)	233 ± 6	22 ± 1	660 ± 20
	Sheet II (n=6)	210 ± 20	21 ± 1	620 ± 40
	**Average (n=9)**	**220 ± 20**	**21 ± 1**	**630 ± 40**

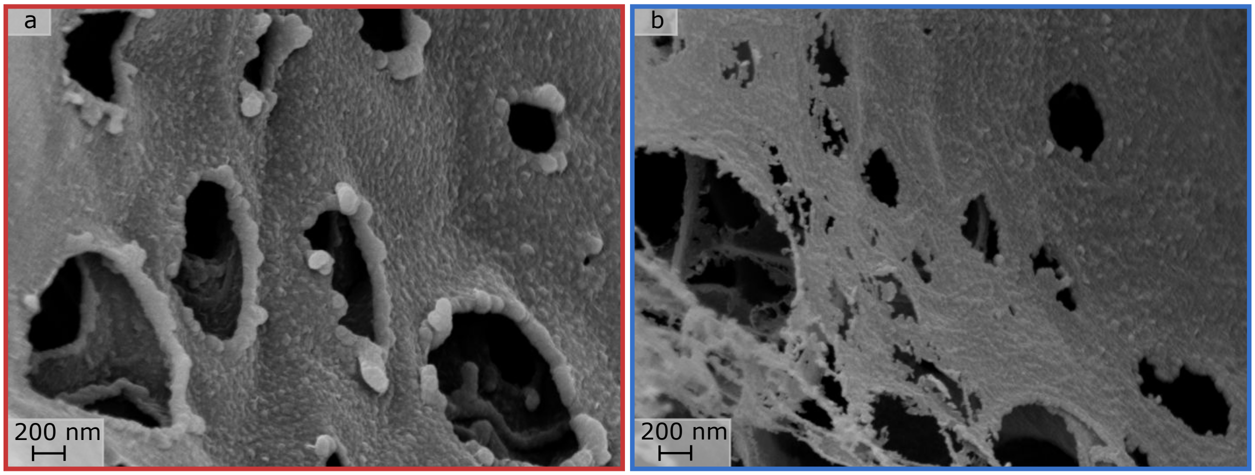

Figure S5. Magnification of 73480 x of the inner pores of LIG A (a) and LIG B (b).

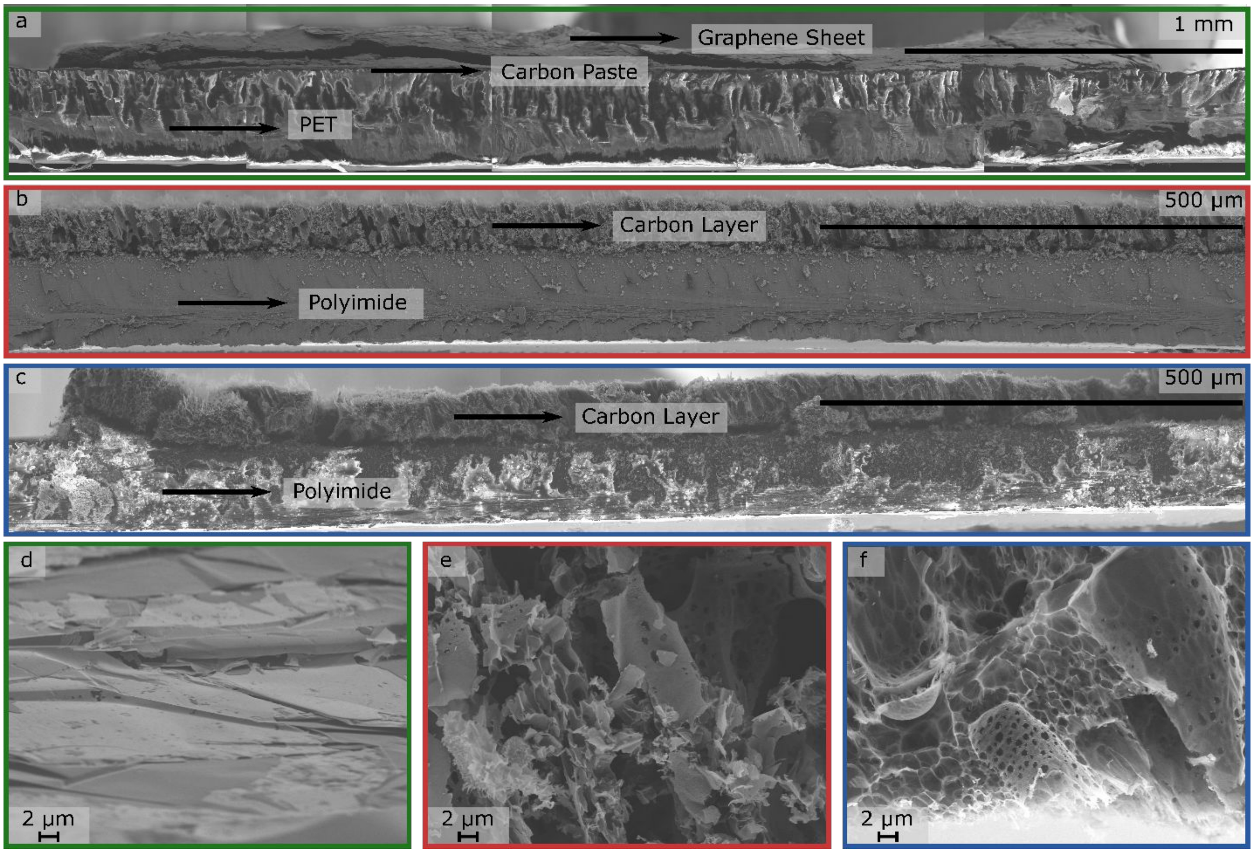

Figure S6. Cross-sectional SEM image of the: a) GS-SPE, showing the PET layer, the carbon paste and the graphene sheet. b) LIG A with the PI layer and the carbon layer. c) LIG B with the PI layer and the carbon layer; Magnified carbon layer on the d) GS-SPE, e) LIG A, and f) LIG B.

Table S6. Summary of Raman spectra peaks.

	**GS-SPE**	**LIG A**	**LIG B**
**D Position (cm^{-1})**	1,347	1,347	1,347
**D Intensity**	36	1,166	894
**D FWHM (cm^{-1})**	1	45	43
**D Area**	854	57,434	38,317
**G Position (cm^{-1})**	1,582	1,575	1,583
**G Intensity**	1,659	1,704	830
**G FWHM (cm^{-1})**	16	45	43
**G Area**	31,235	83,857	39,498
**2D Position (cm^{-1})**	2,710	2,696	2,693
**2D Intensity**	1,644	1,428	673
**2D FWHM (cm^{-1})**	55	73	68
**2D Area**	103,207	115,101	51,176
**D+D' Position (cm^{-1})**	-	2,927	2,928
**D+D' Intensity**	-	158	150
**D+D' FWHM (cm^{-1})**	-	62	63
**D+D' Area**	-	12,018	10,068
**I_D/I_G**	**0.03**	**0.68**	**0.97**
**I_{2D}/I_G**	**3.30**	**1.37**	**1.30**

Table S7. Peak oxidation current difference from the peak current at pH 7 for 50 μM $NaNO_2$.

	Δi_p %		
	**GS-SPE**	**LIG A**	**LIG B**
**pH 6**	-3.60	-3.32	-9.89
**pH 7**	0.00	0.00	0.00
**pH 8**	5.72	-6.14	31.03

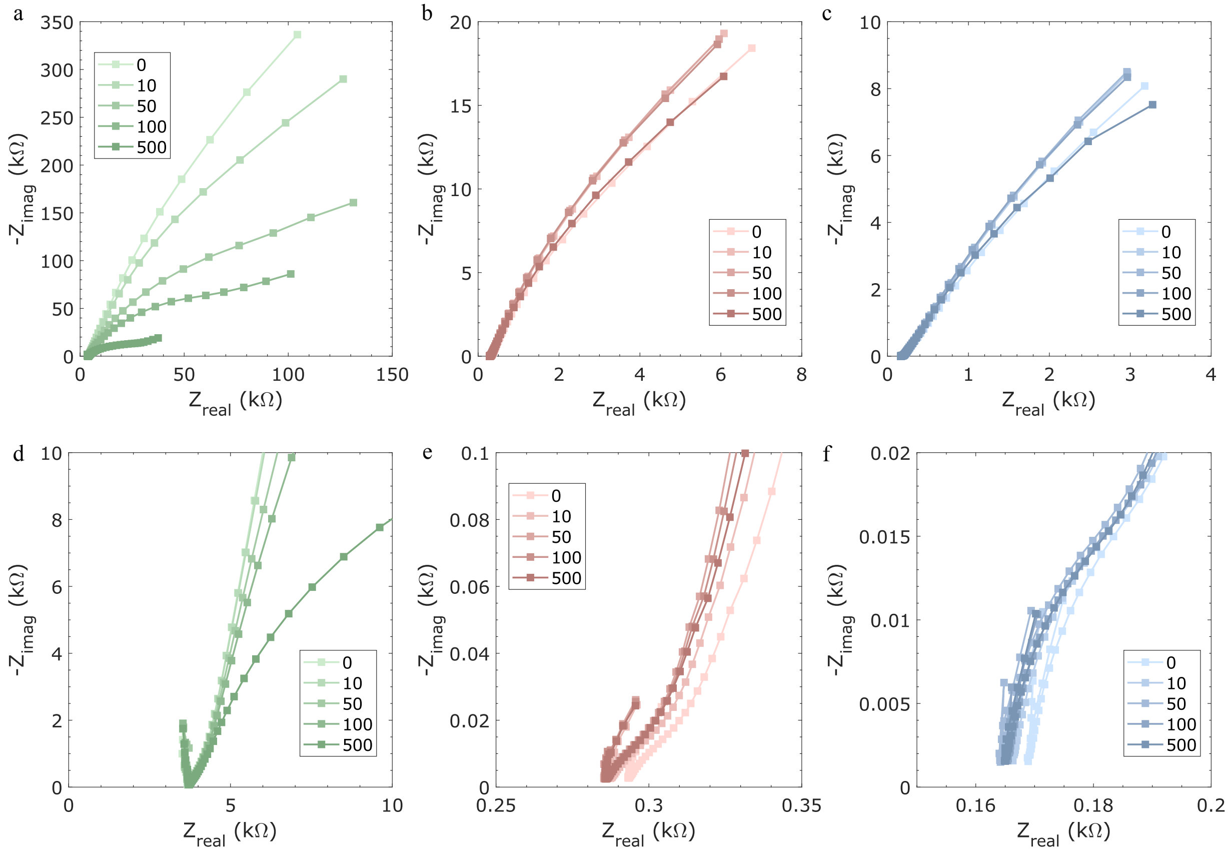

Figure S7. EIS spectra (Nyquist diagrams) with changing concentrations of 10-500 μM $NaNO_2$ in 50 mM NaCl pH 7 for: a) GS-SPE, b) LIG A, and c) LIG B. Zoomed in EIS spectra (Nyquist diagrams) for: d) GS-SPE, e) LIG A, and f) LIG B.

Table S8. Calibration curve parameters based on the DPV measurements of 10-500 µM $NaNO_2$ in 50 mM NaCl pH 7.

	GS-SPE	LIG A	LIG B
**Linear range (µM)**	38-500	10-500	20-500
**Sensitivity (nA/µM)**	5.2 ± 0.3	30 ± 2	40 ± 1
**Intercept (nA)**	197 ± 64	1507 ± 334	2658 ± 220
**R^2**	0.922	0.935	0.985
**RSD (%)**	< 33.6	< 24.5	< 20.6
**LOD (µM)**	37	9	19
